# Supplementary material for: MR1-ligand cross-linking identifies vitamin B6 metabolites as TCR-reactive antigens
Source: Cell Rep Methods. 2025 Aug 4;5(8):101120. doi: 10.1016/j.crmeth.2025.101120 (PMC12461623; doi:10.1016/j.crmeth.2025.101120)
Supplement: Document S1. Figures S1–S5 [file mmc1.pdf]

Cell Reports Methods, Volume *5*

Supplemental information

MR1-ligand cross-linking identifies vitamin B6 metabolites as TCR-reactive antigens

Thierry Schmidlin, Enas Behiry, Hannah Thomas, Garry Dolton, Fabio Marino, Samiul Hasan, Magdalena von Essen, Rose M. Gathungu, Barbara A. Steigenberger, Hayden Selvadurai, Joseph Dukes, Paul E. Brennan, Owen B. Spiller, Jonathan D. Silk, Andrew K. Sewell, and Nicola Ternette

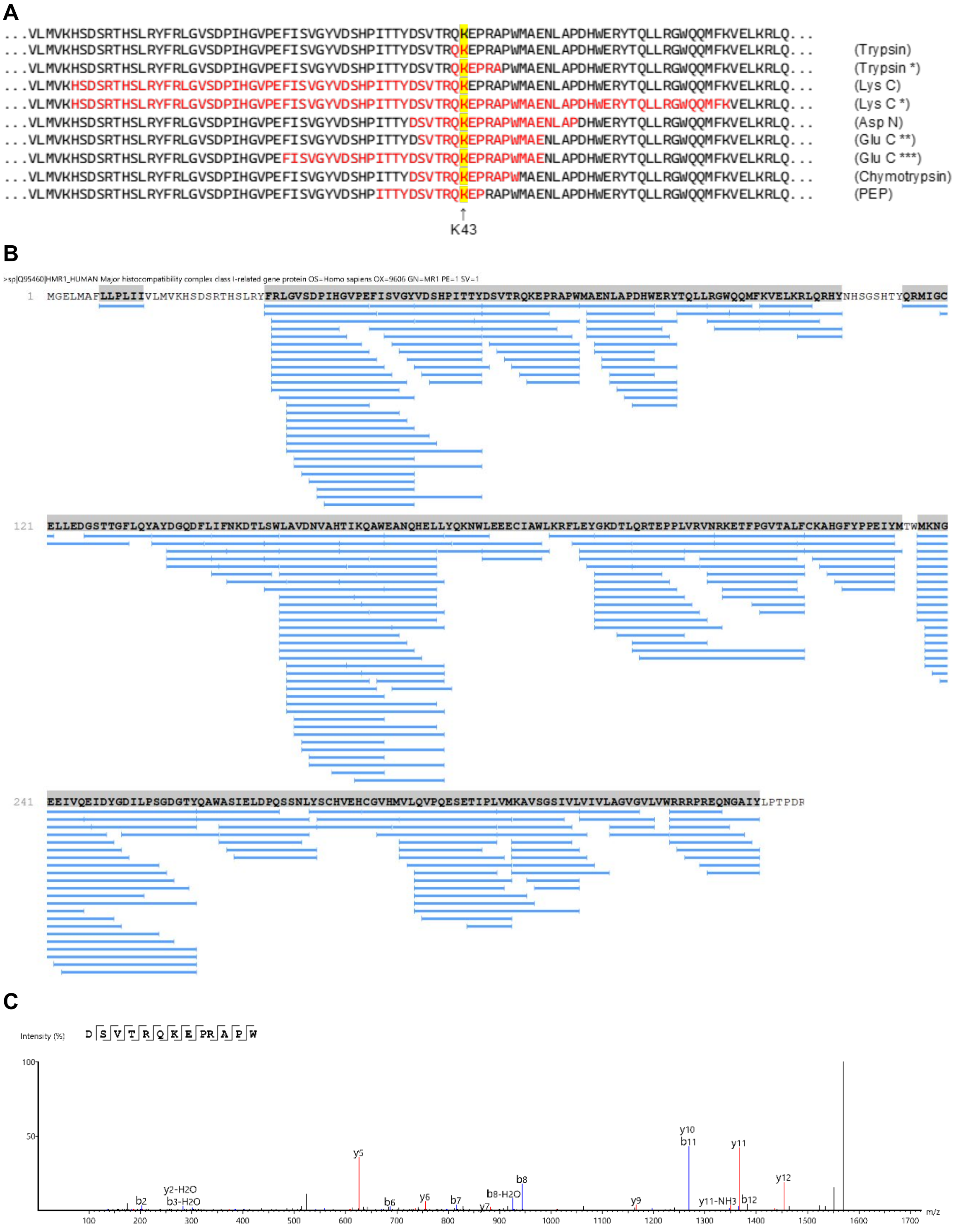

Figure S1. Chymotrypsin digest of MR1, related to Figure 1. (A) *In silico* digestion of MR1 around the K43 residue with different enzymes. Lysine 43 (K43) indicated by yellow highlighting. Predicted digestion products with different enzymes (indicated on the right) in red. * denotes assuming missed cleavage at a ligand modified residue. ** denotes digestion performed in phosphate buffer. *** denotes digestion performed in AMBIC. **(B)** Sequence coverage of MR1 in a typical MR1 enrichment experiment digested by chymotrypsin, measured in data-dependent acquisition mode and analyzed by PEAKS DB. Blue bars

represent peptides identified with a <1% FDR cutoff. **(C)** Exemplary PEAKS DB identification as peptide spectra match of DSVTRQ**K**EPRAPW in the same sample. Sequence-specific y-ions are depicted in red and sequence-specific b-ions in blue. Sequence coverage chart above spectrum indicates presence or absence of all sequence-specific y-ions (vertical bar above sequence indicating presence) and b-ions (vertical bar below sequence indicating presence).

A: PEAKS DB identification of unmodified DSVTRQKEPRAPW

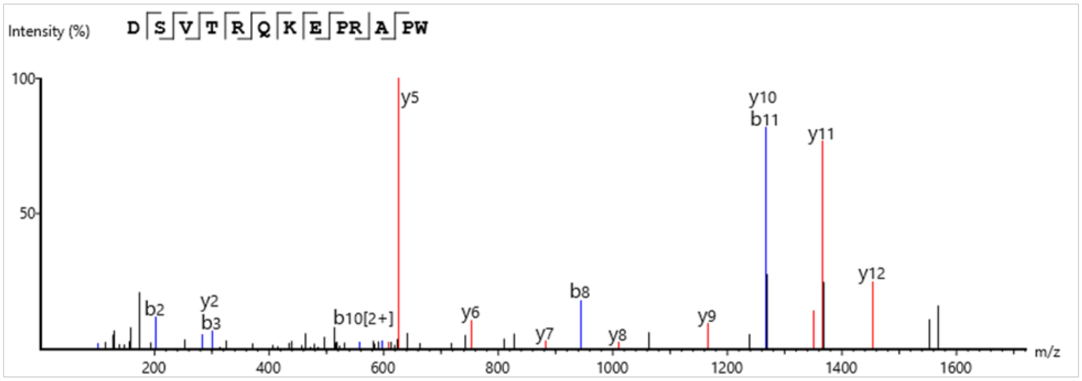

B: PEAKS DB identification of Ac-6-FP linked DSVTRQK(Ac-6-FP)EPRAPW

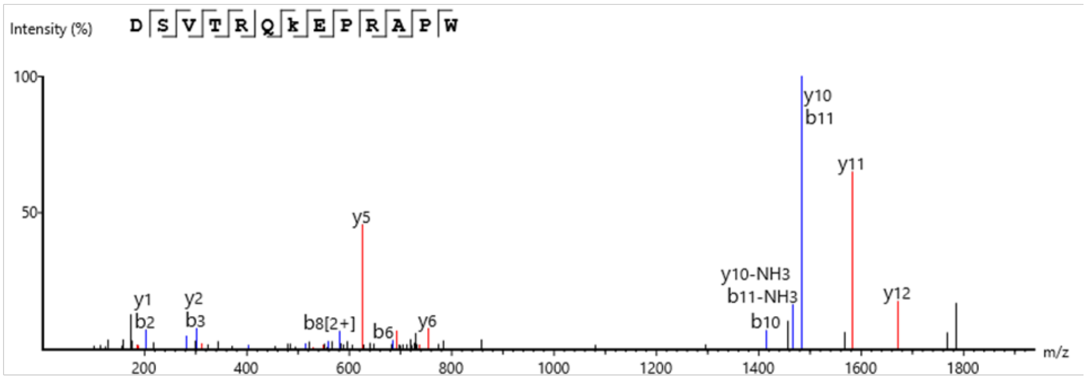

C: Quantitative yield

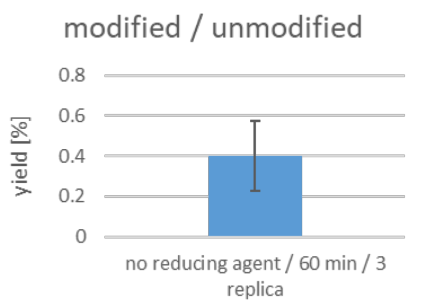

D: Rough yield in the MR1 binding pocket

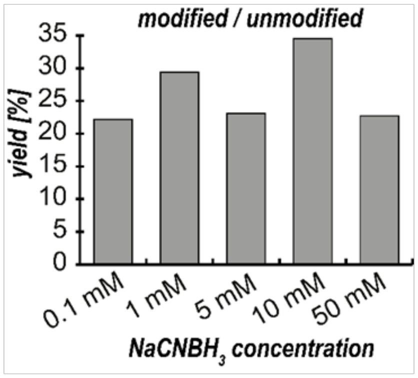

E: PEAKS DB identification of Ac-6-FP linked DSVTRQK(Ac-6-FP)EPRAPW within the MR1 binding pocket

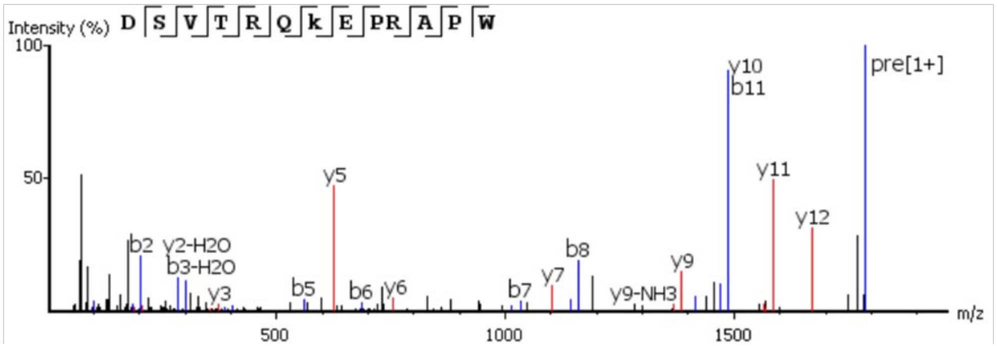

Figure S2. Reductive Amination of Ac-6-FP with synthetic DSVTRQKEPRAPW peptide in the absence of reducing agent and within the MR1-binding pocket, related to Figure 2. (A-C) Peptide to Ac-6-FP crosslinking reaction: Results of mixing equimolar amounts of synthetic DSVTRQ**K**EPRAPW and Ac-6-FP incubated at room temperature for 1 h to assess quantitative yield of background reductive amination happening in the absence of $NaCNBH_3$. **(A)** PEAKS DB identification of non-crosslinked DSVTRQ**K**EPRAPW. **(B)** PEAKS DB identification of Ac-6-FP-crosslinked DSVTRQ**K**(Ac-6-FP)EPRAPW (modified residue indicated in lower case). Sequence-specific y-ions are depicted in red and sequence-specific b-ions in blue. Sequence coverage chart above spectrum indicates presence or absence of all sequence-specific y-ions (vertical bar above sequence indicating presence) and b-ions (vertical bar below sequence indicating presence). **(C)** Quantitative yield determined by signal DSVTRQ**K**(Ac-6-FP)EPRAPW / signal DSVTRQ**K**EPRAPW, displayed in percentage. Signal intensity was determined by LC-MS AUC of 3 most intense isotope signals. Error bars depict standard deviation based on 3 replicates. Yields of ~0.4% were substantially lower than yields observed by adding 0.1mM and 1mM $NaCNBH_3$ (related to Figure 2C). **(D-E)** Reductive amination of Ac-6-FP within the MR1-binding pocket with reducing agent: **(D)** Rough quantitative yield of Ac-6-FP cross-linking in the binding groove at five different concentrations of $NaCNBH_3$ performed at n=1. The crosslinking product was detected at all tested reducing agent concentrations from 0.1 mM to 24 mM. Estimates for the quantitative yields are in the range of ~20-30% for all conditions. **(E)** Peaks DB identification of Ac-6-FP crosslinked DSVTRQ**K**EPRAPW using *in vitro* refolded MR1/β2M/ligand heterotrimer complex. Sequence-specific y-ions are depicted in red and sequence-specific b-ions in blue. Sequence coverage chart above spectrum indicates presence or absence of all sequence-specific y-ions (vertical bar above sequence indicating presence) and b-ions (vertical bar below sequence indicating presence).

A

MSRSVALAVLALLSLSGLEAIQRTPKIQVYSRHPAENGKSNFLNCYVSGFHPSDIEVDLLKNGERI
EKVEHSDLSFSKDWSFYLLYYTEFTPTEKDEYACRVNHVTLSQPKIVKWDRDMGGGGSGGGGS
GGGGSRTHSLRYFRLGVSDPIHGVPEFISVGYVDSHPITTYDSVTRQKEPRAPWMAENLAPDH
WERYTQLLRGWQQMFKVELKRLQRHYNHSGSHTYQRMIGCELLEDGSTTGFLQYAYDGQDFLI
FNKDTLSWLAVDNVAHTIKQAWEANQHELLYQKNWLEEECIAWLKRFLEYGKDTLQRTEPPLVR
VNRKETFPGVTALFCKAHGFYPPEIYMTWMKNGEEIVQEIDYGDILPSGDGTYQAWASIELDPQS
SNLYSCHVEHCGVHMVLQVPQESETIPLVMKAVSGSIVLVIVLAGVGVLVWRRRPREQNGAIYLP
TPDRWSHPQFEK

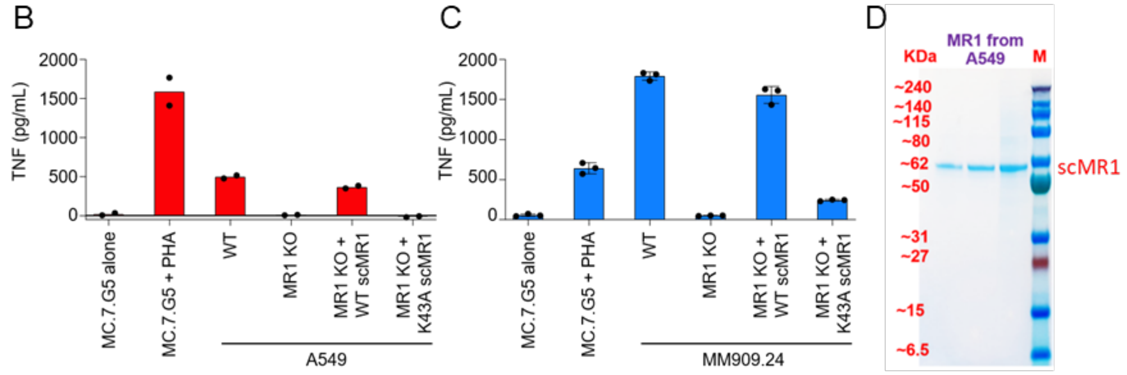

Figure S3. Design, characteristics and purification of scMR1, related to Figure 3. (A) To facilitate further enrichment of MR1 we developed a single chain MR1/β2M (scMR1) construct for expression in various cell lines. Our construct was codon-optimized and designed to express the following protein sequence (GGGGS3 linker highlighted in yellow and strep-II-tag in turquoise). **(B-C)** scMR1 presents ligand to the MC.7.G5 cancer-specific MR1-restricted T-cell clone. Overnight activation assay with T-cell clone MC.7.G5 versus various A549 (panel B) and MM909.24 (panel C) cell lines followed by a TNF ELISA, confirming the functionality of the scMR1 constructs. Individual data plots shown for duplicates (panel B) and error bars depicting standard deviation of triplicate conditions (panel C). **(D)** scMR1 can be recovered from A549 cells with high purity. SDS-PAGE showing purified scMR1 from A549 cells transduced with scMR1 as described in the materials and methods. The three samples represent three consecutive FPLC fractions after Strep-Tactin enrichment eluted with 50 mM biotin. M indicates a lane running molecular size marker. MR1 and β2M were also the most abundant proteins purified from scMR1-K43A expressing MM909.24 cells (related to Figure 3D). We concluded that we could recover strep-II-tagged scMR1 proteins from cell extracts with high purity.

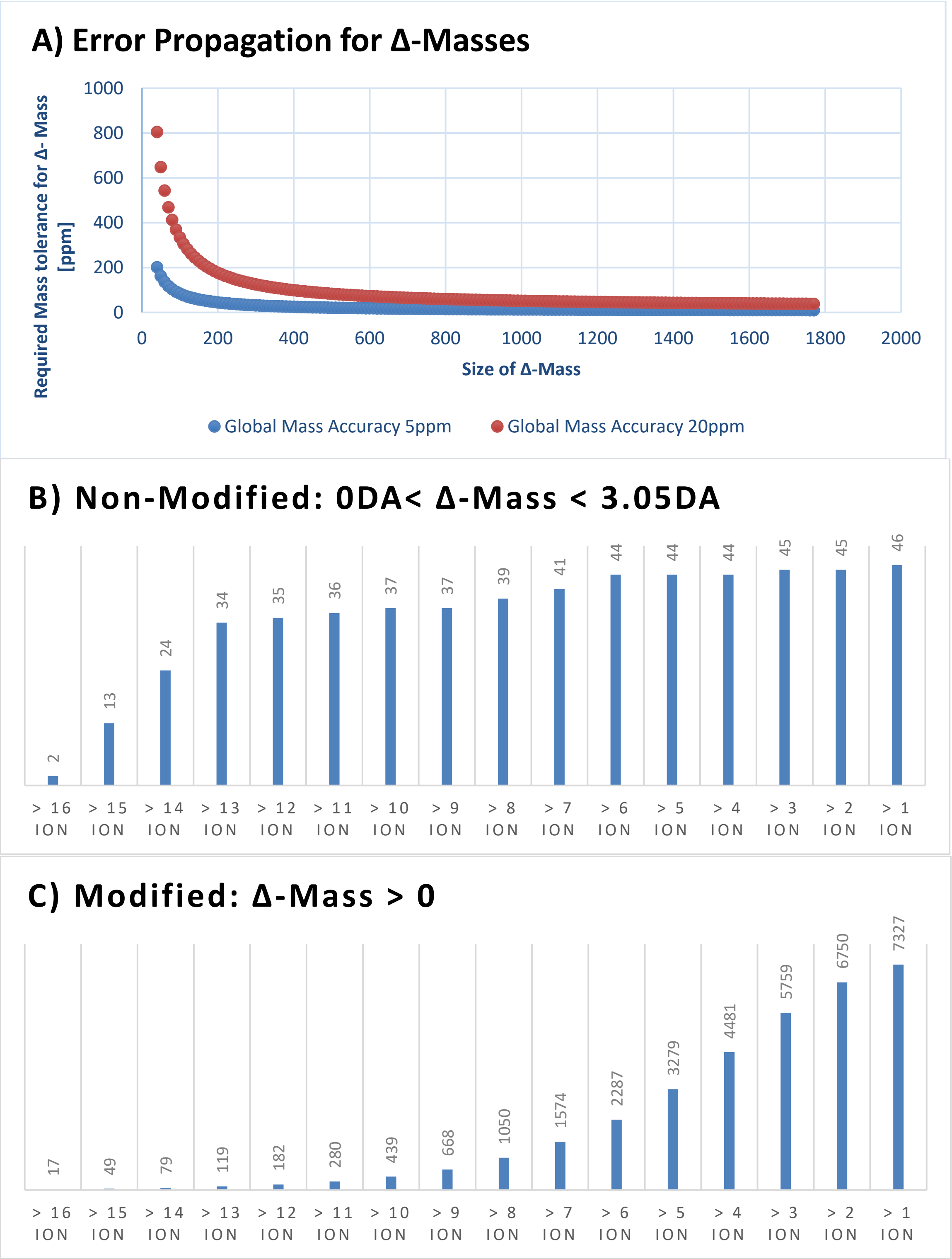

Figure S4. Mass error propagation effects in respect to ligand size and considerations for reporter ion number cut-off values, related to Figure 4. (A) Error propagation as a dependency of absolute ligand size: The graph shows the dependency of the accuracy window for Δ-mass values when calculated from the theoretical peptide mass assuming a constant mass accuracy on the precursor ion measurement level. Blue and red dots indicate the error propagation depending the absolute size of the calculated Δ-mass for an

overall mass accuracy of 5ppm and 20ppm respectively. **(B-C)** Assessing reporter ion cut-off values: Cumulative number of MS/MS spectra in four replicates of A549 cells, where a specific number of reporter ions were found. **(B)** Cumulative number of spectra within any of the Δ-mass ranges [0-0.05Da], [0.95-2.05Da] [1.95-2.05Da], [2.95-3.05Da], representing unmodified DSVTRQKEPRAPW and its isotopologues (closed search). We expected this approach to pinpoint all MS/MS spectra of DSVTRQ**K**EPRAPW that were also identified as DSVTRQ**K**EPRAPW in the database search. In this analysis we observed that the number of hits associated with DSVTRQ**K**EPRAPW reached a plateau in the range of 10 -11 reporter ions per spectrum. **(C)** Cumulative number of spectra with a Δ-mass higher than 0Da (open search). Here, the number of hits grew continuously when lowering the required number of reporter ions. The observed discrepancy between open (Δ-mass >3.5 Da) and closed (0 < Δ-mass < 3.5Da) searches was explained by the additional specificity provided by the narrowly defined precursor mass, whereas the open search was prone to be increasingly affected by random matches upon decreasing reporter ion stringency. Hence, the observed plateau could be used to reach an informed choice about reasonable reporter ion number cut-offs which was set to ≥10 ions for subsequent analyses.

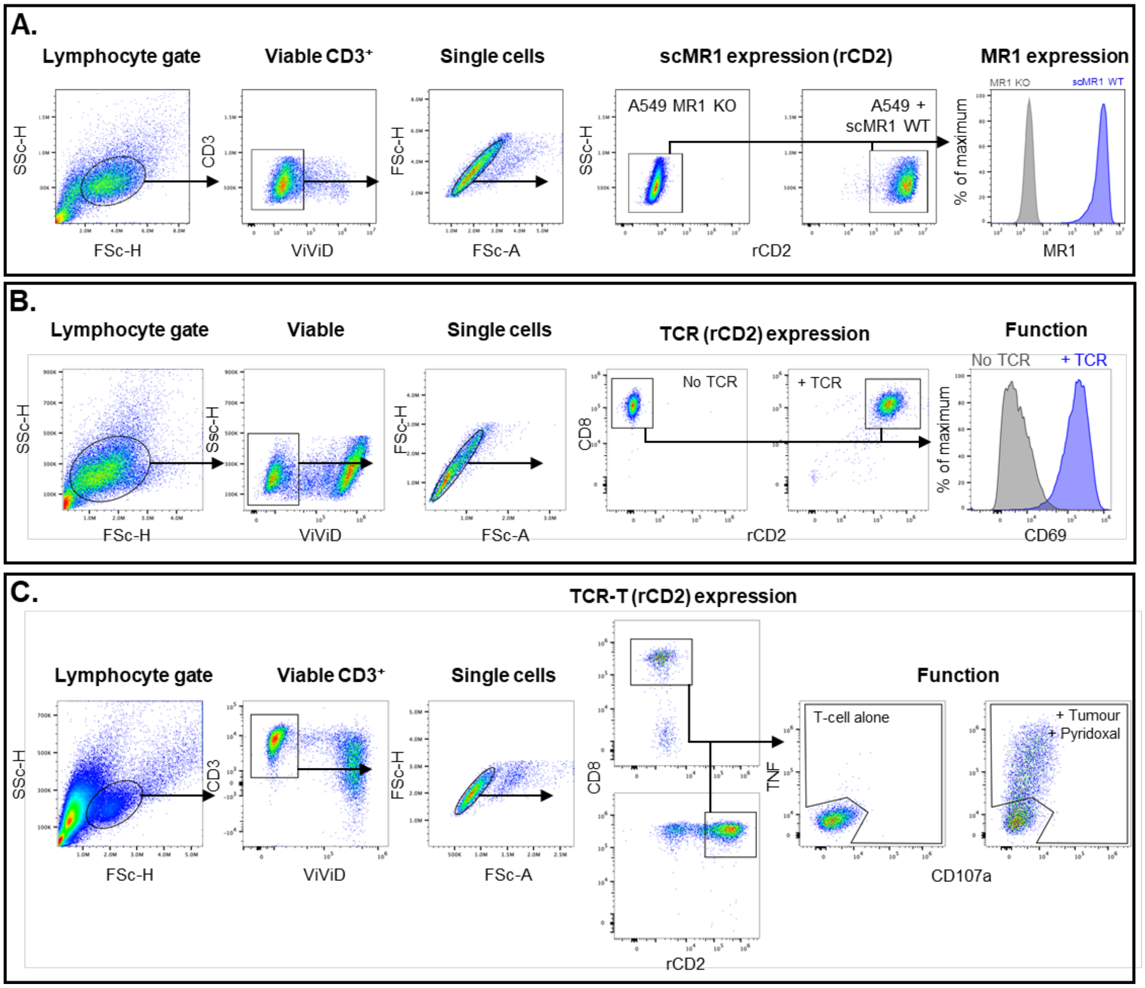

Figure S5. Flow cytometry gating strategies, related to Figure 5 and Figure 6. (A) MR1 antibody staining; A549 and A549 MR1 KO cells did not express rCD2 but A549 cells with scMR1 -WT or -K43A were gated on rCD2 as a co-marker, then stained with the MR1 surface antibody. **(B)** CD69 assay; Triple Parameter Reporter (TPR; $CD8^+$) Jurkat cells were gated on $rCD2^+$ for TCR transduced cells then stained with the CD69 surface antibody. **(C)** T107 assay; cells were gated on $rCD2^+$/$CD8^+$ for A-F7 TCR-T or just $CD8^+$ for the untransduced T cells, then TNF^+ versus $CD107a^+$ for reactivity.
