# Supplementary material for: MR1-ligand cross-linking identifies vitamin B6 metabolites as TCR-reactive antigens
Source: Cell Rep Methods. 2025 Aug 4;5(8):101120. doi: 10.1016/j.crmeth.2025.101120 (PMC12461623; doi:10.1016/j.crmeth.2025.101120)
Supplement: Document S2. Article plus supplemental information [file mmc7.pdf]

Cell Reports
Methods

Article

# MR1-ligand cross-linking identifies vitamin B6 metabolites as TCR-reactive antigens

## Graphical abstract

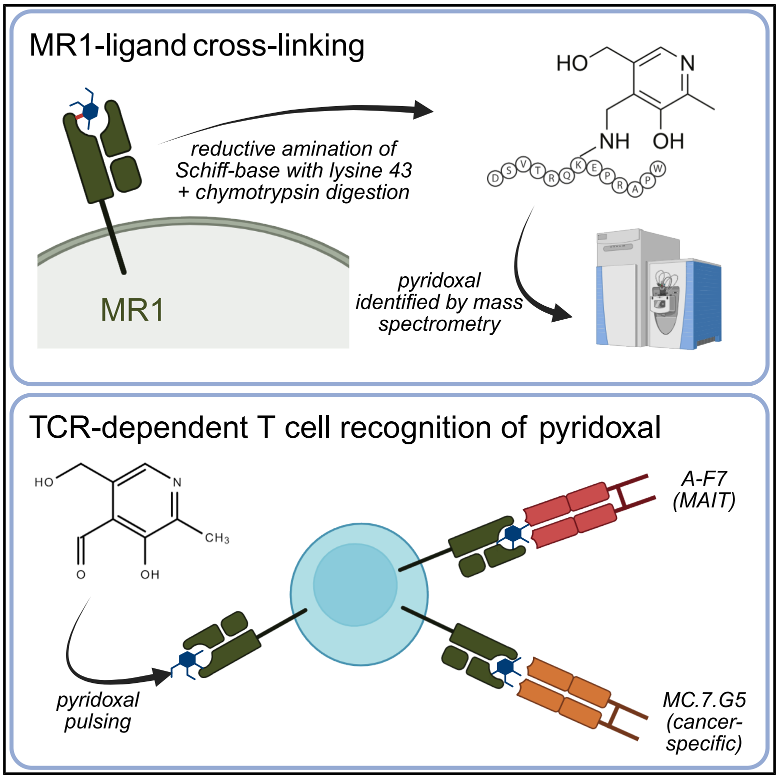


## Highlights

- Workflow for identification of MR1 metabolite ligands via cross-linking LC-MS/MS
- Identification of vitamin B6 vitamers pyridoxal and PLP as MR1 ligands
- Validation of upregulated MR1 surface expression on ligand-pulsed cells
- TCR-dependent T cell recognition of pyridoxal and PLP-pulsed cells via MR1

## Authors

Thierry Schmidlin, Enas Behiry, Hannah Thomas, ..., Jonathan D. Silk, Andrew K. Sewell, Nicola Ternette

## Correspondence

schmidlt@uni-mainz.de (T.S.),
sewellak@cardiff.ac.uk (A.K.S.),
nternette001@dundee.ac.uk (N.T.)

## In brief

Schmidlin et al. present a method for the discovery of metabolite antigens presented by major histocompatibility complex class I-related protein (MR1) by chemical cross-linking and subsequent mass spectrometry analysis. Using this approach, the authors successfully identified the B6 vitamers pyridoxal and pyridoxal 5′-phosphate as naturally occurring, immunogenic MR1 ligands.

Schmidlin et al., 2025, Cell Reports Methods 5, 101120
August 18, 2025 © 2025 The Author(s). Published by Elsevier Inc.
https://doi.org/10.1016/j.crmeth.2025.101120

CellPress

Cell Reports Methods

CellPress
OPEN ACCESS

Article

# MR1-ligand cross-linking identifies vitamin B6 metabolites as TCR-reactive antigens

Thierry Schmidlin,[1,2,3,13,*] Enas Behiry,[4,13] Hannah Thomas,[4] Garry Dolton,[4] Fabio Marino,[5,6] Samiul Hasan,[4] Magdalena von Essen,[5] Rose M. Gathungu,[5] Barbara A. Steigenberger,[7] Hayden Selvadurai,[5] Joseph Dukes,[5] Paul E. Brennan,[8,9] Owen B. Spiller,[4] Jonathan D. Silk,[5] Andrew K. Sewell,[4,10,11,14,*] and Nicola Ternette[3,12,14,15,*]

[1]Institute of Immunology, University Medical Center of the Johannes Gutenberg-University Mainz, Mainz, Germany
[2]Research Center for Immunotherapy (FZI), University Medical Center of the Johannes-Gutenberg University, Mainz, Germany
[3]Nuffield Department of Medicine, Centre for Immuno-Oncology, Oxford OX3 7DQ, UK
[4]Division of Infection and Immunity, Cardiff University School of Medicine, Cardiff CF14 4XN, UK
[5]Enara Bio Ltd., The Bellhouse Building, Oxford Science Park, Sanders Road, Oxford OX4 4GD, UK
[6]Biomolecular Mass Spectrometry and Proteomics, Utrecht Institute of Pharmaceutical Science, University of Utrecht, Utrecht, the Netherlands
[7]Mass Spectrometry Core Facility, Max Planck Institute of Biochemistry, Martinsried, Germany
[8]Centre for Medicines Discovery and NIHR, Oxford Biomedical Research Centre, Centre for Medicines Discovery, Nuffield Department of Medicine, University of Oxford, Oxford OX3 7FZ, UK
[9]Alzheimer's Research UK, Oxford Drug Discovery Institute, Centre for Medicines Discovery, Nuffield Department of Medicine, University of Oxford, Oxford OX3 7FZ, UK
[10]Systems Immunology Research Institute, Cardiff University, Cardiff, Wales, UK
[11]Division of Infection and Immunity, Kumamoto University, Kumamoto, Japan
[12]University of Dundee, School of Life Sciences, Dow Street, Dundee, Scotland DD1 5EH, UK
[13]These authors contributed equally
[14]These authors contributed equally
[15]Lead contact
*Correspondence: schmidlt@uni-mainz.de (T.S.), sewellak@cardiff.ac.uk (A.K.S.), nternette001@dundee.ac.uk (N.T.)
https://doi.org/10.1016/j.crmeth.2025.101120

**MOTIVATION** The conserved antigen presentation molecule major histocompatibility complex class I-related protein 1 (MR1) presents ligands to T cells that signify intracellular bacteria or cancerous transformation. A lack of methodology for trapping and identifying MR1-bound ligands from the cell surface has limited our understanding of immunogenic metabolite antigens. Here, we describe an approach for antigen discovery based on chemically cross-linking ligands to MR1.

## SUMMARY

Major histocompatibility complex class I-related protein 1 (MR1) plays a central role in the immune recognition of infected cells and can mediate T cell detection of cancer. Knowledge of the nature of the ligands presented by MR1 is still sparse and has been limited by a lack of efficient approaches for MR1 ligand discovery. Here, we present a cross-linking strategy to investigate Schiff base-bound MR1 ligands. Our methodology employs reductive amination to stabilize the labile Schiff base bond between MR1 and its ligand, allowing for the detection of ligands as covalent MR1 adducts by mass spectrometry-based proteomics. We apply our approach to identifying vitamin B6 vitamers pyridoxal and pyridoxal 5′-phosphate (PLP) as MR1 ligands and show that both compounds are recognized by T cells expressing either A-F7, a mucosal-associated invariant T (MAIT) cell T cell receptor (TCR), or MC.7.G5, an MR1-restricted TCR reported to recognize cancer cells, highlighting them as immunogenic MR1 ligands.

## INTRODUCTION

Antigen presentation on the cell surface by major histocompatibility complex (MHC) class I-related protein 1 (MR1) is a vital part of mammalian immune surveillance. A series of seminal papers in 2012–2014 by the McCluskey and the Rossjohn groups showed that MR1 can present products and derivatives of the microbial vitamin B2 and B9 biosynthesis


© 2025 The Author(s). Published by Elsevier Inc.
This is an open access article under the CC BY license (http://creativecommons.org/licenses/by/4.0/).

pathways to a subset of T cells called mucosal-associated invariant T (MAIT) cells.[1–3] Since then, MR1 has been suggested to play a role in the immune response to bacterial infection and is now known to be a central molecule involved in immune recognition and control of infectious diseases due to its ability to present metabolites to T cells. MR1 is also thought to be able to present ligands at the surface of cancer cells.[4,5] Amino acid residue K43 in the MR1 antigen-binding groove plays a key role in presenting some MR1 ligands by forming a covalent bond via Schiff base formation between the primary amine residue of the lysine and an aldehyde or ketone moiety of the ligand.[5,6] K43 also performs a key role in retaining MR1 in the endoplasmic reticulum (ER) prior to ligand capture.[7]

Efforts have been made to refine our understanding of the nature and extent of the self- and non-self-MR1-presented metabolome, primarily using screening and MR1-metabolite refolding approaches,[8] soluble MR1 expression systems coupled to metabolomics,[9] or *in silico* prediction protocols.[10,11] These efforts have resulted in less than twenty compounds being classified as *bona fide* or potential MR1 ligands and T cell antigens to date, including products and derivates of the vitamin B2 and B9 pathways.[12] Recently, carbonyl adducts of nucleobases were found to be self-antigens presented by MR1 using a mass spectrometry (MS) approach. These ligands were found to be immunogenic and able to activate MR1-restricted T cells.[13] Furthermore, the repertoire of mycobacterial ligands was expanded by Krawic et al., who described that hydroxyindolyl-ribityllumazine isoforms can differentially activate MAIT cells.[14] Tools for the systematic recovery of ligands bound to MR1 in cells, however, are still lagging behind equivalent methods routinely used to identify MHC-bound peptides.[15]

Here, we present an MS-based method to investigate MR1-bound antigens at the cell surface of cancer cells. Our approach leverages the characteristic Schiff base formation between the ligand and the MR1 lysine 43 residue (K43), which is known to play a crucial role in the binding of several known MR1 ligands either directly at the ligand's aldehyde group (e.g., 6-formylpterin[1] or acetyl-6-formylpterin[16] [Ac-6-FP]) or through the use of small modifiers such as glyoxal or methylglyoxal (e.g., 5-(2-oxopropylideneamino)-6-D-ribitylaminouracil [5-OP-RU][3]). Using reductive amination chemistry, we transform the labile Schiff base into a stable secondary amine, generating a covalent metabolite-protein cross-link[17,18] that facilitates the subsequent screening of the MR1-presented antigens as molecular mass shifts observed using a standard bottom-up proteomics liquid chromatography-tandem MS (LC-MS/MS) measurement. We used this approach to identify the candidate ligands pyridoxal and pyridoxal 5′-phosphate (PLP) as presented by MR1 on A549 lung cancer cells. We subsequently validated these vitamin B6 vitamers as *bona fide* MR1 ligands by demonstrating that they induce cell surface upregulation of MR1. Exogenous pyridoxal and, to a lesser degree, PLP were shown to activate T cells expressing the MAIT T cell receptor (TCR) A-F7 and the MR1-restricted, cancer-reactive MC.7.G5 TCR,[4] suggesting a potential role of these ligands in MR1-mediated immune recognition.

## RESULTS

### Reductive amination leads to cross-linking of the potent MR1 ligand Ac-6-FP to synthetic DSVTRQKEPRAPW peptide

Aiming to employ reductive amination chemistry as a tool to perform *de novo* MR1 ligand discovery, we designed a conceptual workflow, depicted in Figure 1, that consists of 8 steps: (1) sample lysis, (2) high-specificity MR1/β2M enrichment, (3) MR1/ligand cross-linking by Schiff base reduction, (4) proteolytic digestion, (5) bottom-up LC-MS/MS analysis, (6) detection of ligand masses by mass shift analysis, (7) candidate ligand generation, and (8) biochemical validation.

Key considerations were the choice of digestion enzyme and reducing agent used. *In silico* digests were performed to assess the best proteolytic peptide to study MR1 K43 modifications (Figure S1A). The chymotryptic 13-mer DSVTRQ**K**EPRAPW (MR1 D37-W49, lysine 43 indicated in bold text) proved to be the most promising candidate in terms of peptide length. DSVTRQ**K**EPRAPW showed excellent LC-MS performance in its ligand-free form (Figures S1B and S1C) and was therefore used for all subsequent experiments. Sodium cyanoborohydride ($NaCNBH_3$) was chosen as a reducing agent based on its known characteristics of being highly specific to Schiff base reduction, resulting in limited off-target reactions.[17,18]

The initial feasibility of the ligand/MR1 cross-linking reaction was demonstrated by incubating synthetic DSVTRQ**K**EPRAPW with the well-characterized MR1 ligand Ac-6-FP (1:1) and $NaCNBH_3$ at various concentrations (0.1 [based on Holden et al.[18]], 1, and 24 [based on Boersema et al.[17]] mM) and reaction times (5, 10, 30, and 60 min). We were able to detect the expected Ac-6-FP/peptide cross-linking product in several conditions both (1) by unambiguous identification via database searches against the entire UniProt database performed by PEAKS using Ac-6-FP cross-linking as a variable modification (Figure 2A) and (2) by generating extracted ion chromatograms (XICs) of the expected molecular mass (Figure 2B). We estimated rough quantitative yields of the reaction by comparing the chromatographic area under the curve (AUC) values of Ac-6-FP-bound DSVTRQ**K**EPRAPW versus unmodified DSVTRQ**K**EPRAPW, reaching maximum values of roughly 8%. Counterintuitively, in this experiment, we observed higher quantitative cross-linking yields when using lower concentrations of reducing agents (Figure 2C). We speculate that this may be the result of an overreaction of $NaCNBH_3$ at higher concentrations that causes either a further reduction of the Ac-6-FP/peptide cross-linking product, resulting in lower amounts of the expected cross-linking product, or a reduction of Ac-6-FP to a product that cannot form Schiff bases anymore prior to the cross-linking. In support of the latter, we saw a substantial decay of the LC-MS signal of non-cross-linked Ac-6-FP when using higher concentrations of $NaCNBH_3$ (Figure 2D). However, a definite reaction product could not be identified. Conversely, incubation of the peptide with Ac-6-FP for 60 min without the addition of $NaCNBH_3$ resulted in substantially lower cross-linking yields of ~0.4% (Figures S2A–S2C).

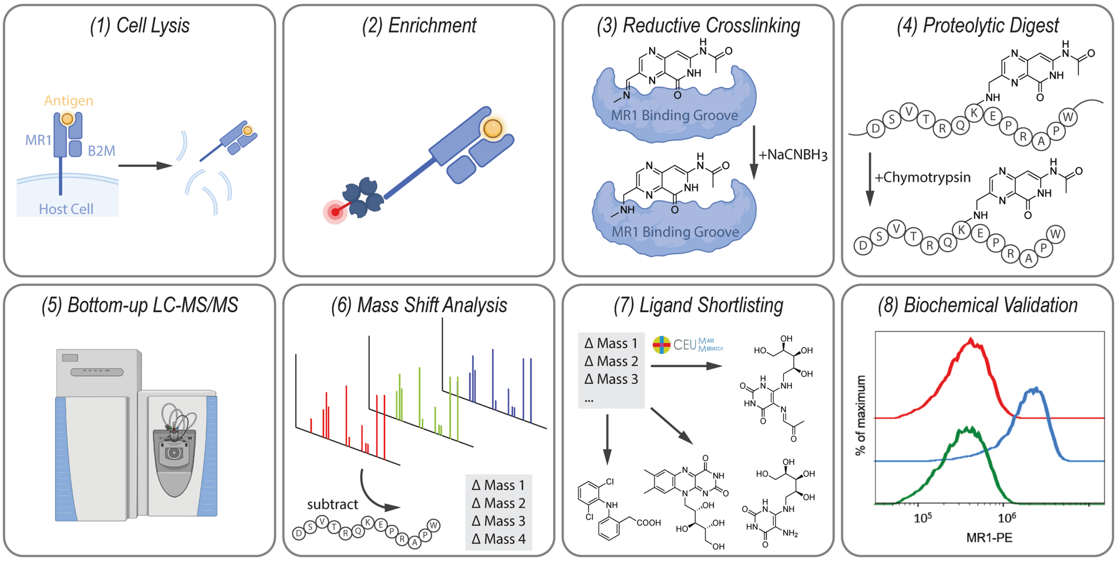


**Figure 1. Workflow for MR1-dependent antigen discovery by protein-metabolite cross-linking**

(1) Cells presenting antigens on MR1 were lysed, followed by (2) strep-tag-based enrichment of MR1 from the lysate. (3) The unstable Schiff base that formed between the ligand and the MR1 K43 residue was subsequently stabilized using reductive amination followed by (4) proteolytic digest, giving rise to the K43-specific DSVTRQ**K**EPRAPW peptide (Figure S1). (5) Peptide samples were analyzed by bottom-up LC-MS/MS. (6) Data were scoured for evidence of variable modification on the MR1-specific K43 peptide DSVTRQ**K**EPRAPW using a combination of mass shift analysis and peptide sequence-specific reporter ions. (7) Mass shifts of potential ligands were shortlisted according to their potential chemical composition and (8) validated biochemically by their ability to induce MR1 surface expression in cells, as measured by flow cytometry. See also Figure S1.

## Identification of the Ac-6-FP-modified peptide DSVTRQKEPRAPW from refolded and Ac-6-FP-loaded MR1 protein after reductive amination and enzymatic release

We next confirmed that we could detect the ligand-modified peptide in the context of an *in vitro* refolded MR1/β2M/ligand heterotrimer complex. We utilized diclofenac-loaded MR1, a previously identified MR1-binding compound that does not form a Schiff base with K43,[19] and performed a ligand exchange reaction with a 500-fold excess of Ac-6-FP prior to the reduction reaction (Figure 2E). After chymotrypsin digestion, we were able to identify the Ac-6-FP-linked peptide with high accuracy (Figures 2F, S2D, and S2E) and with higher yields than during our optimization experiments with the synthetic peptide, reaching ∼30%.

## Reductive amination allows covalent cross-linking of MR1 ligands in cultured cells

We next set out to evaluate the applicability of our approach for capturing MR1 ligands from cells. We discovered that isolating high yield and purity of MR1 was essential for our approach in order to obtain the desired sensitivity required to detect ligand-bound DSVTRQ**K**EPRAPW by LC-MS/MS. Initial tests using the MR1-specific antibody 26.5 for MR1 immunoprecipitations from Jurkat cells and the MR1-overexpressing cell line C1R.MR1 did not yield sufficient MR1 (Table S1).[20,21] We therefore developed a recombinant platform to express fully functional, C-terminally strep-II-tagged single-chain MR1*01/β2M (scMR1) molecules with either the wild-type (WT) lysine or—as a negative control—alanine at position 43 (Figures 3A and S3A). These scMR1 constructs were expressed in A549 cells and MM909.24 cells, where the endogenous MR1 genes had been knocked out by CRISPR-Cas9.[4,22] Cell surface expression of scMR1-WT and scMR1-K43A in A549 and MM909.24 cells is shown in Figure 3B. We confirmed the full functionality of scMR1 by showing that scMR1 could function to present both MAIT-recognized bacterial ligands and MC.7.G5-recognized cancer ligands[4] to relevant TCRs (Figures 3C, S3B, and S3C for MC.7.G5). The strep-II-tag allowed highly specific enrichment of scMR1 protein, as demonstrated by SDS-PAGE gel analysis (Figure S3D). This was further confirmed using LC-MS/MS, where MR1 and β2M were identified as the most abundant proteins in a preparation of streptavidin-purified proteins from scMR1-K43A-transduced MM909.24 cells, reaching a sequence coverage of 57% and 60%, respectively (Figure 3D). Pulsing the scMR1-transfected MM909.24 cells with 50 μM Ac-6-FP for 16 h in a single replicate proof-of-concept experiment and subjecting them to our protocol allowed us to confirm the detection of the Ac-6-FP-linked DSVTRQ**K**EPRAPW on MM909.24 cells, reaching a quantitative yield of almost 10% (Figure 3E).

## Development of a reporter ion-based analysis pipeline enables detection of MR1 ligands

When attempting to analyze the LC-MS/MS data for potential K43-associated mass shifts following the addition of Ac-6-FP, we encountered a deficiency of available, appropriate software tools. Common proteomics data analysis software packages, such as MSFragger[23] and MaxQuant,[24] suffer from the generally low size assumed for unspecific posttranslational

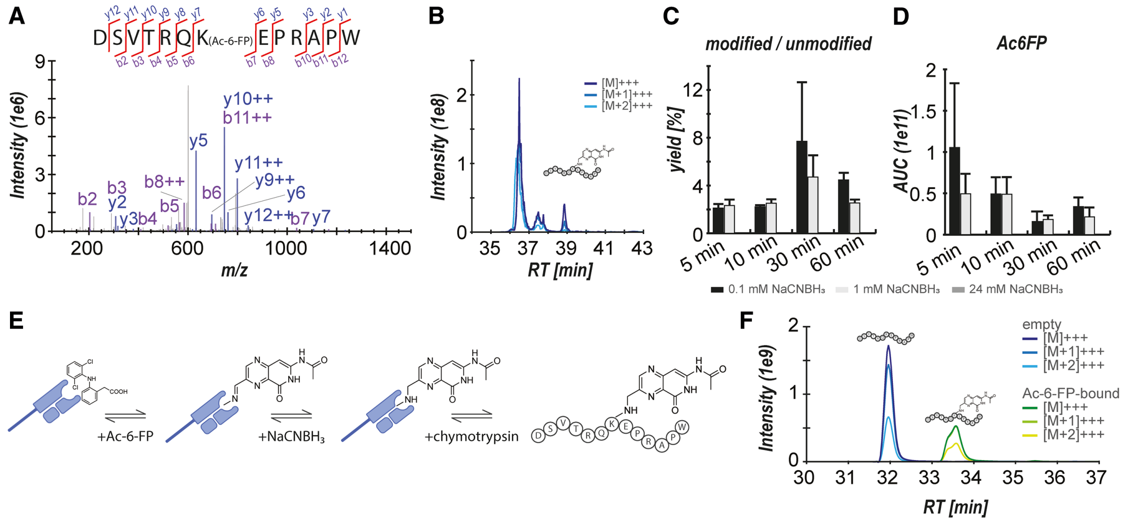


**Figure 2. Reductive amination of Ac-6-FP with synthetic DSVTRQKEPRAPW peptide and within the MR1-binding pocket**

(A–D) Proof of concept for cross-linking reaction using synthetic DSVTRQ**K**EPRAPW and the well-established MR1 ligand Ac-6-FP *in vitro*.

(A) Representative spectrum from triplicate analysis for a peptide identification by PEAKS defining Ac-6-FP as a variable modification. Sequence-specific b-ions (purple) and y-ions (blue) are annotated in the spectra and visualized in the peptide sequence. Red lines pointing to the right above the peptide sequence indicate the presence of a position-specific y-ion, and red lines pointing to the left below the peptide sequence indicate presence of a position-specific b-ion.

(B) Representative extracted ion chromatograms (XICs) of the Ac-6-FP-bound DSVTRQ**K**EPRAPW precursor, including the three most abundant isotopes (M, M+1, and M+2). The horizontal axis represents chromatographic retention time (RT) in minutes (min).

(C) Reaction yields were assessed by calculating ratios of the chromatographic area under the curve (AUC) of Ac-6-FP-bound DSVTRQ**K**EPRAPW and unmodified DSVTRQ**K**EPRAPW at various reaction conditions, suggesting an improved yield at lower concentrations of $NaCNBH_3$. Experiments were performed in triplicate, with error bars indicating standard deviations.

(D) Similarly, AUCs for free Ac-6-FP decrease at higher $NaCNBH_3$ concentrations, indicating the possibility of an off-target reduction reaction happening in increased reducing conditions prior to cross-link formation. Experiments were performed in triplicate, with error bars indicating standard deviations.

(E and F) Proof of concept for cross-linking reaction within the MR1 binding groove.

(E) Diclofenac-loaded recombinant MR1/β2M complexes were incubated with Ac-6-FP to induce ligand exchange and perform reductive amination at 5 different $NaCNBH_3$ concentrations.

(F) Representative XICs for the reaction product after chymotryptic digestion in comparison to the non-cross-linked peptide. The product was detected in all five experimental conditions tested. See also Figure S2.

modifications and a low sensitivity due to employing a proteome-wide search space rather than a focus on one specific binding residue, which can only partly be resolved by reduced database sizes. We therefore established our own data analysis pipeline based on the presence of reporter ions, such as sequence-specific y-ions (C-terminal fragment ions) and b-ions (N-terminal fragment ions), that are unaffected by the cross-linked ligand (Figure 3F). Individual fragment spectra were shortlisted based on the presence of those reporter ions and further criteria, such as noise levels, visual spectral quality, and similarity to spectra observed for unmodified DSVTRQ**K**EPRAPW. The masses of the K43-associated ligands were calculated for each spectrum individually based on the difference between the experimentally determined peptide mass and the theoretical mass of unmodified DSVTRQ**K**EPRAPW (Δ-mass, listed in Table S2). We further used the Centro de Estudios Universitarios (CEU) mass mediator tool to compute candidate elemental compositions for all experimentally determined K43-associated mass shifts and assigned those elemental compositions with candidate compounds of potential ligands.[25] Within a <2 min retention time difference from the unmodified DSVTRQ**K**EPRAPW, signals were observed corresponding to the expected molecular weights of the known MR1-binding ligand 6-formylpterin, likely as a degradation product of Ac-6-FP (Figure 3G; Table S3), albeit at a much lower intensity. Furthermore, at an even lower intensity than 6-formylpterin, we observed a signal that potentially corresponded to methylglyoxal (Figure 3H). The low intensity observed for other ligands is consistent with previous studies showing that Ac-6-FP can outcompete other MR1 ligands[26] and did not allow a robust quantitative assessment.

## *De novo* antigen discovery in A549 cancer cell lines in culture leads to the discovery of two MR1 ligand candidates

To demonstrate the capability of our approach to perform *de novo* identification of MR1-presented metabolite antigens, we examined scMR1 from stably transduced A549 cells where the natural *MR1* genes had been ablated.[22] We assessed the cutoff values for the required number of reporter ions according to the spectral qualities obtained for unmodified DSVTRQ**K**EPRAPW (Figure S4), eventually retaining all spectra with 10 or more reporter ions (Table S4). These spectra were further visually shortlisted, providing a resource for potential molecular masses of MR1 ligands that can be mined for candidate compounds. We

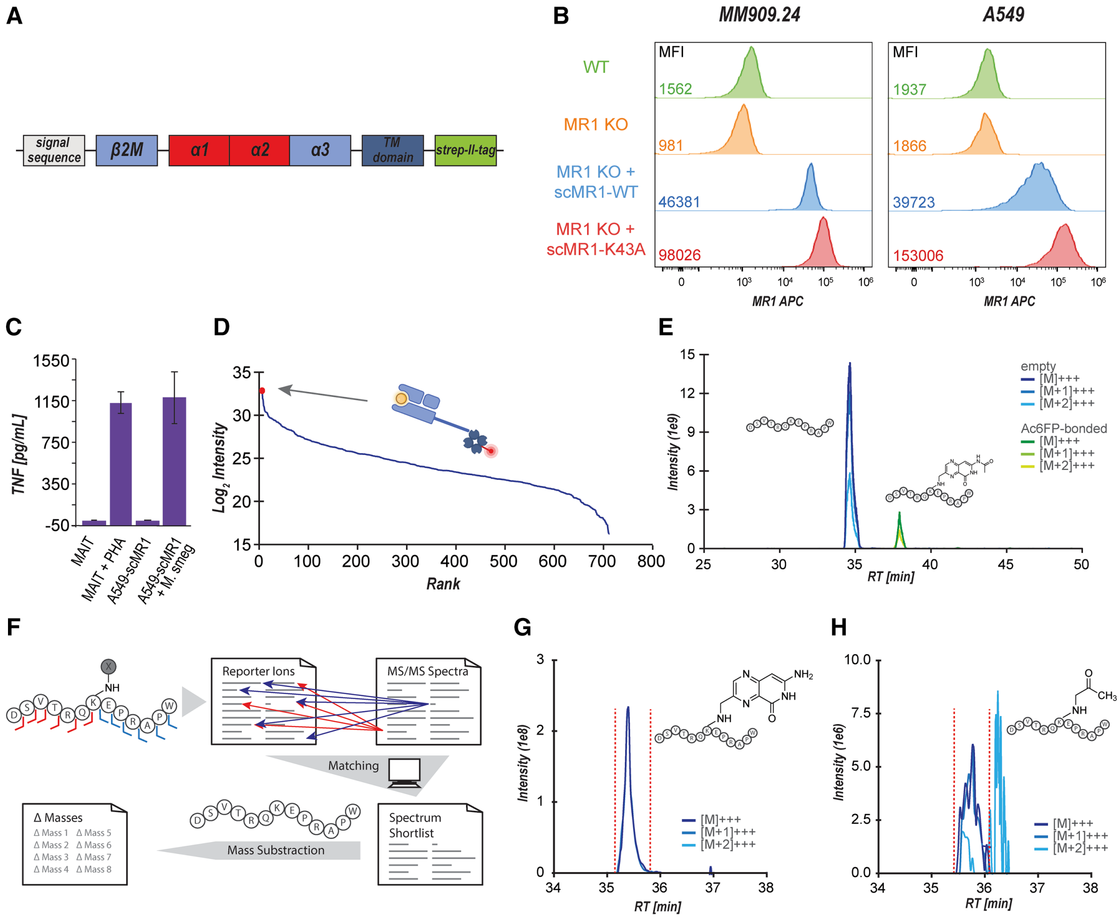


**Figure 3. Development of an enrichment strategy for MR1-dependent antigen discovery by protein-metabolite cross-linking and *de novo* MR1 antigen discovery**

(A) Schematic of a recombinant platform to express fully functional, C-terminally-tagged single-chain MR1/β2M (scMR1) molecules with either lysine or alanine at position 43, developed for high-specificity MR1 enrichment. The alpha 1, 2, 3, and transmembrane (TM) domains of MR1 are depicted.

(B) MR1 staining of A549 (left) and MM909.24 (right) cell lines, either wild type (WT), MR1 knockout (MR1 KO), and MR1 KO cells transduced with scMR1 (MR1 KO + scMR1-WT) and mutant scMR1 (MR1 KO + scMR1-K43A). Numbers in the left-hand corner are the MR1-specific Allophycocyanin (APC) mean fluorescence intensities (staining with anti-MR1 26.5 antibody clone). Gates are set for viable, single cells.

(C) Overnight activation assay with MAIT cell TCR-T (primary $CD8^+$ T cells transduced with A-F7 MAIT TCR) versus *M. smegmatis*-infected and uninfected A549 cells followed by a tumor necrosis factor (TNF) ELISA confirming MAIT cell recognition of scMR1 in the presence of endogenous antigen. Error bars depict the standard deviation of duplicate conditions.

(D) Enrichment efficiency obtained from MM909.24 cells stably transduced with scMR1-K43 molecules based on protein abundances obtained by LC-MS/MS (MR1 and β2M are highlighted separately as red dots that overlap).

(E) Proof of concept for the detection of MR1/Ac-6-FP cross-link in a cell-based system. scMR1-transfected MM909.24 melanoma cells pulsed with 50 μM Ac-6-FP for 16 h were subjected to the cross-linking workflow. The graph shows extracted ion chromatograms for DSVTRQ**K**EPRAPW and DSVTRQ**K**EPRAPW bound to Ac-6-FP, respectively, including the three most abundant isotopes (M, M+1, and M+2) for each of the two peptide variants.

(F) Schematic of the data analysis workflow employed to detect DSVTRQ**K**EPRAPW cross-linked to unknown ligands. Peptide sequence ladder ions unaffected by the ligand (y1–y6 and b1–b6) were used as reporter ions. MS/MS spectra containing these ions were subsequently shortlisted. Subtraction of the theoretical DSVTRQ**K**EPRAPW peptide mass generates Δ-mass values for cross-linked ligands that can be corrected for the mass of the free ligand, which can be queried for candidate compounds using tools such as CEU mass mediator.

(G and H) Applied to scMR1-transfected MM909.24 pulsed with 50 μM Ac-6-FP for 16 h, the data analysis pipeline successfully detected spectra indicating the presence of other ligands bound to MR1, such as 6-formylpterin (XIC, G) and methylglyoxal (XIC, H).

See also Figure S3 and Tables S2 and S3.

demonstrated this approach by shortlisting two candidate molecules, guided by CEU mass mediator: pyridoxal (Δ-mass = 151.06; Figure 4) and PLP (Δ-mass = 231.01). These molecules stood out on the candidate list, as both are vitamers of vitamin B6 and are known to exhibit a propensity to form Schiff bases.[27] Moreover, both compounds comprise aromatic systems, potentially enabling π-stacking in the MR1 binding groove, a common feature of the majority of the MR1 ligands known to date.[12]

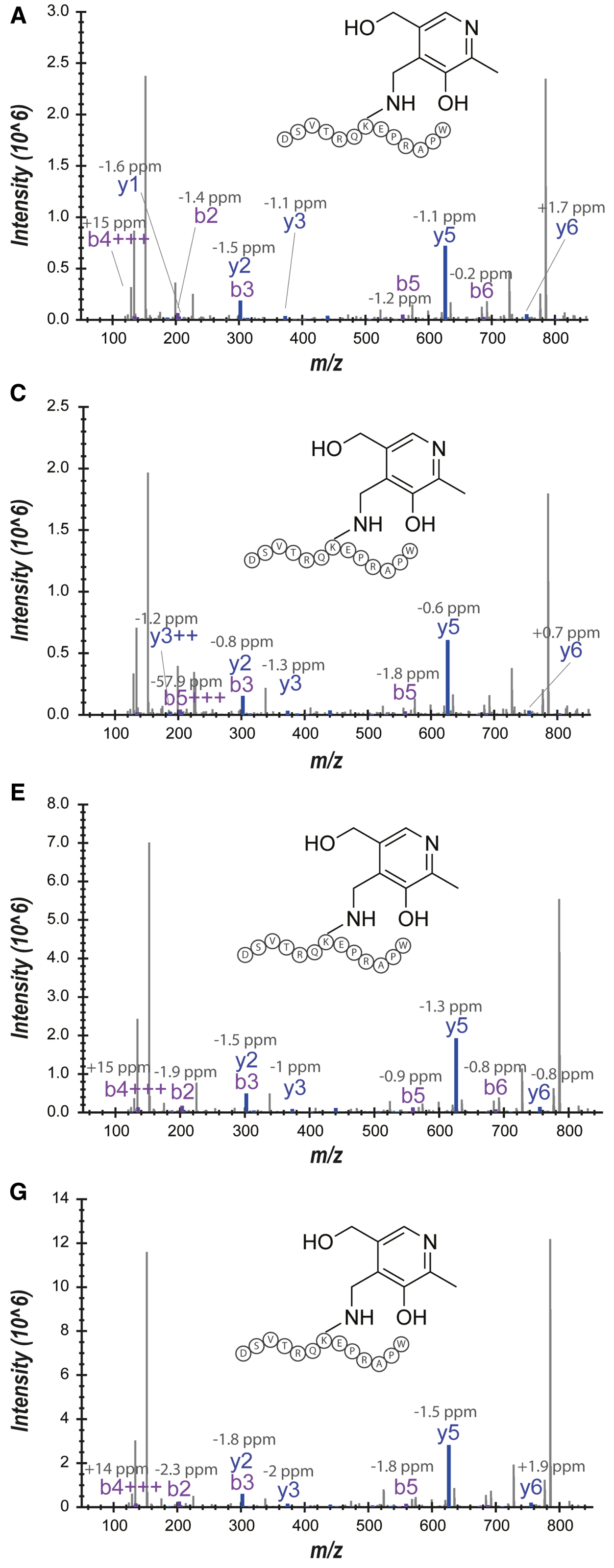


(legend on next page)

## Pyridoxal upregulates MR1 surface expression

We tested the capacity of pyridoxal and PLP to induce MR1 surface expression in scMR1-transfected A549 cells (Figures 5A, 5B, and S5; Tables S5A–S5D). Pyridoxal significantly ($p = 4.5 \times 10^{-6}$, ANOVA with Tukey's post hoc test) increased MR1 surface expression compared to baseline surface expression, corroborating its role as an MR1 ligand. We next verified that the pyridoxal-induced increase in MR1 surface expression was not an artifact of our recombinant, overexpressed scMR1 construct using A549 cells expressing WT MR1, again observing a significant ($p = 2.1 \times 10^{-2}$) pyridoxal-induced increase in MR1 surface expression (Figures 5C and 5D). Both experiments also showed a subtle increment of MR1 surface expression upon pulsing with PLP, which reached significance in scMR1-expressing A549 cells ($p = 1.7 \times 10^{-3}$) yet remained non-significant in WT A549 cells.

## Pyridoxal is an MR1-restricted T cell ligand

We next investigated whether pyridoxal and PLP could act as TCR agonists. Triple-reporter Jurkat T cells, which lack β2M and surface expression of MR1,[28] that were transfected with the A-F7 MAIT TCR[29] were shown to recognize *M. smegmatis*-infected A549 cells as a positive control (Figure 6A; Table S5E). These cells were also able to recognize A549 cells incubated with 1–100 μg/mL pyridoxal in parallel with recognition of both *M. smegmatis* and pyridoxal being dependent on MR1 expression. Having established that pyridoxal could act as an agonist ligand for the A-F7 MAIT TCR, we next examined how T cells expressing this TCR responded to PLP. Titrations of pyridoxal and PLP showed that pyridoxal was the more potent agonist ($EC_{50}$ = 1.14 μM [0.54–1.68] compared to >30 μM for PLP) for Jurkat.MAIT.A-F7 cells (Figure 6B; Tables S5F and S5G). We next confirmed that pyridoxal could act as an agonist for primary $CD8^+$ T cells from three donors that were transduced with the A-F7 TCR (Figure 6C; Table S5H). We further extended the potential relevance of pyridoxal as an MR1 ligand by showing that this ligand could activate T cells expressing the MC.7.G5 TCR (Figure 6D; Tables S5I and S5J). We conclude that pyridoxal can act as a ligand for known MR1-restricted TCRs.

## DISCUSSION

Rather than using recombinant protein refold assays or soluble MR1 expression systems for the identification of MR1 ligandomes, we sought to capture ligands that are naturally presented by MR1 on the cellular surface directly using a chemical cross-linking approach. In contrast to refolding assays, our approach does not require *a priori* knowledge about the nature of these ligands. Furthermore, the focus on a stable, covalent linkage between K43 and the ligand provides direct evidence of the MR1-to-ligand interaction.

PLP can be synthesized from pyridoxine, pyridoxamine, and pyridoxal. These B6 vitamers are essential to mammalian metabolism and need to be acquired through the diet. The microbiome may also provide a direct source for these molecular components in humans, as bacteria and yeast can synthesize these compounds *de novo*. In our cellular experiments, pyridoxine was supplied in cell culture media and can be transformed by the cell into PLP by pyridoxal kinase (PDXK) after cellular uptake. PLP, in turn, can be directly converted to pyridoxal by pyridoxal phosphate phosphatase PHOSPHO2. Pyridoxal has been quantified in human serum at low nanomolar levels,[30] and these levels are variable depending on the nutritional uptake of vitamin B6 vitamers.[31] The addition of 10% bovine serum to cell culture media provides a further likely source of B6 vitamers in our experiments.

We demonstrated that pyridoxal pulsing stabilizes and upregulates both MR1 and scMR1 in A549 cells but not K43 mutant MR1, validating the stabilization of MR1 by pyridoxal in a K43-dependent manner. Results from pulsing experiments with PLP were less conclusive, and a slight but significant upregulation was observed in scMR1-expressing A549 cells but not in WT A549. We cannot exclude spontaneous hydrolysis of PLP to pyridoxal or enzymatic conversion of the two vitamers, which might affect MR1 surface expression; however, we conclude that PLP is a less potent MR1 ligand. Importantly, our evidence showing that pyridoxal and PLP bound to lysine 43 of scMR1-WT expressed in A549 cells shows that these B6 vitamers act as MR1 ligands without the requirement for exogenous application at supraphysiologic concentrations. Although not a primary aim of our study, we also examined whether pyridoxal and PLP could act as TCR agonists in the context of MR1. The addition of excess exogenous pyridoxal at 1–100 μg/mL was able to induce the activation of Jurkat T cells and primary $CD8^+$ T cells transduced with the MR1-restricted MAIT TCR A-F7. Pyridoxal ($EC_{50}$ ~1 μM) was more potent than PLP ($EC_{50}$ ~34 μM) at activating Jurkat cells expressing the A-F7 TCR. Exogenous pyridoxal could also act as an agonist of the MC.7.G5 TCR, which has been reported to recognize cancer cells.[4]

PLP is an essential cofactor for DNA and amino acid metabolism.[32] Interestingly, it has been shown in the context of numerous patients with cancer that pyridoxal levels in plasma are lower than in healthy individuals and that the cancer depletes vitamin B6 from the blood by feeding its higher metabolic demand.[33] In support of these observations, PDXK, the enzyme that converts pyridoxine into PLP (see above), has been identified as upregulated in non-small cell lung carcinoma (NSCLC) and therefore resembles a therapy-independent prognostic marker in patients with NSCLC.[34] A potential link between suppressed tumor development upon high vitamin B6 intake and enhanced cell-mediated immunity has been posited as early as 1987 in the context of (H238) cell-induced tumors in mice.[35] While entirely speculative at this stage, enhanced uptake of

**Figure 4. Discovery and validation of pyridoxal as MR1 ligand**
(A and B) scMR1-transfected A549 cells were subjected to reductive cross-linking, shortlisting pyridoxal as a candidate ligand. Data are shown for (A) the annotated fragment spectrum for K43-bound pyridoxal (sequence-specific b- and y-ions are depicted in purple and blue, respectively, in the spectra, with their respective mass error in ppm depicted above) and (B) the corresponding extracted ion chromatogram.
(C–H) Corresponding data for replicates 2–4.
See also Figure S4 and Table S4.

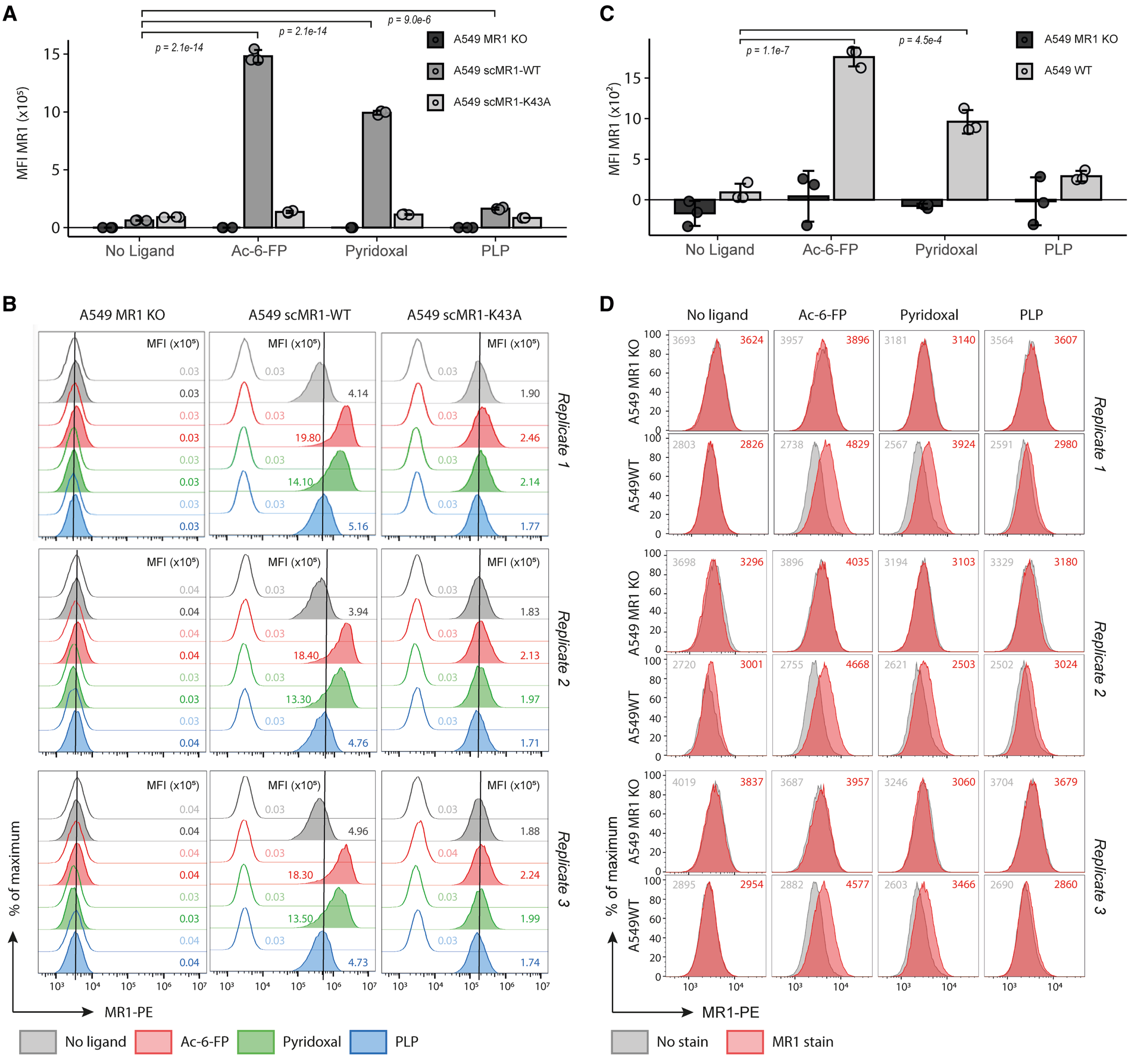


**Figure 5. Ligand-induced upregulation of MR1 surface expression**

(A and B) Pyridoxal upregulates overexpressed scMR1 at the cell surface. The A549 MR1 KO cell line with scMR1-WT or scMR1-K43A was treated with two candidate compounds at 100 μg/mL overnight at 37°C and 5% $CO_2$ and stained with MR1-specific pycoerythrin (PE)-conjugated antibody (MR1-PE).

(A) Mean fluorescence intensity (MFI) of MR1 staining. rCD2 was used as a co-marker for scMR1 and used for gating during flow cytometry. MFI without MR1 antibody has been subtracted from the MFI with MR1 antibody. Triplicate conditions are shown, with error bars depicting standard deviation (Table S5A). *p* values displayed are adjusted *p* values obtained using one-way ANOVA followed by Tukey's honest significant difference (HSD) post hoc test to correct for multiple comparisons (Table S5B).

(B) Flow cytometry for all data displayed in (A), including no-stain condition, showcasing reproducibility across replica. Displayed are all of the triplicate conditions. The vertical line has been set at the no-ligand condition to aid visualization.

(C and D) Pyridoxal upregulates naturally expressed MR1 at the cell surface. A549 WT and A549 MR1 KO cell lines were treated with candidate compounds at 100 μg/mL overnight at 37°C and 5% $CO_2$ and stained with MR1-PE antibody.

(C) MFI of MR1 staining. MFI without MR1 antibody has been subtracted from the MFI with MR1 antibody. Triplicate conditions are shown, with error bars depicting standard deviation (Table S5C). *p* values displayed are adjusted *p* values obtained using one-way ANOVA followed by Tukey's HSD post hoc test to correct for multiple comparisons (Table S5D).

(D) Flow cytometry for all data displayed in (C). Displayed are all of the triplicate conditions with MFI for no stain (gray) and MR1 stain (red).

See also Figure S5 and Table S5.

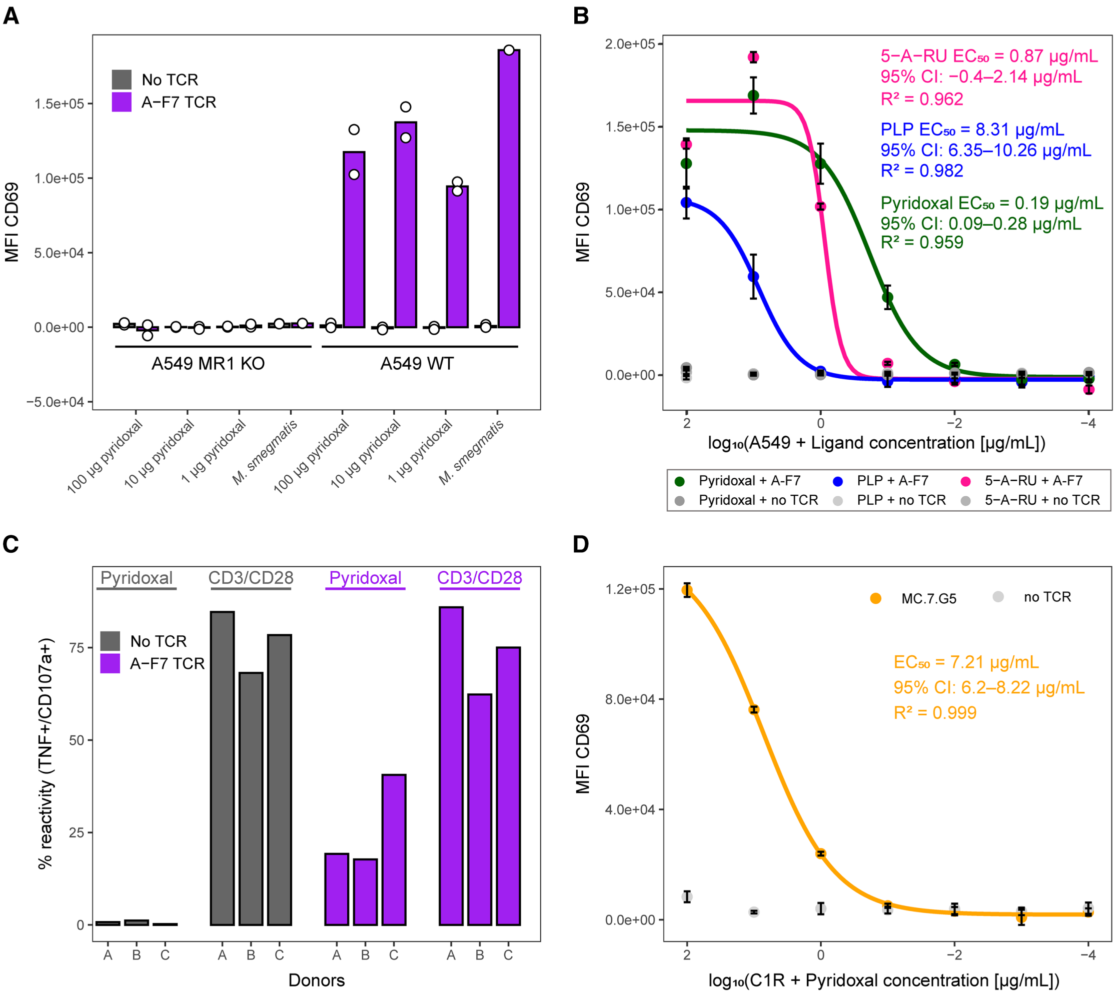


**Figure 6. The B6 vitamers pyridoxal and PLP activate Jurkat cells expressing the A-F7 MAIT TCR and the MC.7.G5 TCR, as well as primary CD8$^+$ T cells expressing the A-F7 MAIT TCR**

(A) Jurkat cells with no TCR or transduced with A-F7 MAIT TCR were co-incubated overnight with A549 WT and A549 MR1 KO cell lines treated with pyridoxal at 100, 10, and 1 μg/mL or loaded with *M. smegmatis* (MOI: 1:300). Cells were stained for CD69 expression with mean fluorescence intensity (MFI) displayed. Background MFI of Jurkat cells with A549 WT or MR1 KO alone with no pyridoxal or *M. smegmatis* was subtracted. Jurkat cells with A-F7 were gated on co-marker rCD2$^+$. Data display duplicate conditions (Table S5E).

(B) Jurkat cells with no TCR or transduced with A-F7 MAIT TCR were co-incubated overnight with A549 WT and the following compounds: 5-A-RU (converts to MAIT ligand 5-OP-RU in cells and was added in the absence of exogenously applied methylglyoxal, which increases potency), pyridoxal and PLP at 100, 10, 1, 0.1, 1 × $10^{-2}$, 1 × $10^{-3}$, and 1 × $10^{-4}$ μg/mL. Cells were stained for CD69 expression with MFI displayed. Background MFI of Jurkat cells with A549 cells alone with no pyridoxal was subtracted. Jurkat cells expressing the A-F7 TCR were gated on the rCD2 co-marker. Assay was performed in triplicate (Table S5F), and curves were fitted using a four-parameter logistic model. Points indicate mean values, with error bars depicting standard deviation. $EC_{50}$ values with a 95% confidence interval (CI) and $R^2$ are indicated, with the results reproducible over two assays (Table S5G).

(C) Primary CD8$^+$ T cells from three healthy donors with no TCR transduction or expression of the A-F7 TCR to generate TCR-T cells, were co-incubated for 4 h with A549 WT cells ± pre-treatment with 100 μg/mL of pyridoxal, and then reactivity measured via T107 assay. T cells were also incubated alone or with CD3/CD28 Dynabeads, with the latter acting as a positive control. Cells were gated on lymphocytes, viable CD3$^+$, single cells, rCD2$^+$/CD8$^+$ (or CD8$^+$ for the untransduced), and then TNF$^+$ versus CD107a$^+$ for reactivity. For the pyridoxal conditions, background reactivity toward A549 cell lines with no pyridoxal has been subtracted. For reactivity toward CD3/CD28 Dynabeads, the reactivity for the T cell-alone condition has been subtracted (Table S5H).

(D) Jurkat cells with no TCR or transduced with MC.7.G5 TCR were co-incubated overnight with C1R cells ± pyridoxal at 100, 10, 1, 0.1, and 1 × $10^{-2}$ μg/mL. Cells were stained for CD69 expression with MFI displayed. Background MFI of Jurkat cells alone with no pyridoxal was subtracted. Jurkat cells with MC.7.G5 TCR were gated on co-marker rCD2$^+$. Assay was performed in triplicate (Table S5I), and curves were fitted using a four-parameter logistic model. Points indicate mean values, with error bars depicting standard deviation. $EC_{50}$ values with a 95% CI and $R^2$ are indicated (Table S5J).

See also Figure S5 and Table S5.

vitamin B6, as well as altered levels and activation states of enzymes responsible for intracellular vitamer B6 conversion in cancer cells, could affect the levels of pyridoxal and PLP availability and propensity as MR1 ligands, which will be an exciting topic for further investigation.

During the review process for this study, McInerney et al. published a paper demonstrating that MR1 presents vitamin B6-related compounds for recognition by MR1-restricted T cells.[36] Their methodology involved purifying MR1 from cells, followed by heat denaturation to release bound ligands, which were then analyzed via MS. Unlike our approach, which preserves the covalent interaction between MR1 and its ligands, the method used by McInerney et al. sought to distinguish true ligands from nonspecific interactors by comparing ligand abundance in MR1 purified from cells overexpressing either MR1 or $MR1^{K43A}$ and from cells overexpressing MR1 pulsed with Ac-6-FP. Using this approach, McInerney et al. identified pyridoxal as a candidate ligand and verified this via protein crystallography, and the findings revealed the formation of a Schiff base between MR1 K43 and the aldehyde group of pyridoxal, corroborating our results. Interestingly, although McInerney et al. did not detect PLP in their ligand screen, they included it in validation experiments where co-crystallization confirmed Schiff base formation between K43 and PLP in the MR1 binding groove.[36] McInerney et al. further showed that T cells expressing the MC.7.G5 TCR were activated by pyridoxal presented by antigen-presenting cells expressing MR1*01 and MR1 carrying the Arg9His ($MR1^{R9H}$) polymorphism found in the MR1*04 allele, which occurs in less than 1% of the population.[37] In addition, T cells expressing the A-F7 TCR responded to plate-bound $MR1^{R9H}$-pyridoxal complexes. Structures of MAIT AF-7 TCR in complex with MR1-pyridoxal and MR1-PLP were also reported (PDB: 9CGR and 9CGS). Notably, McInerney et al. found that cancer cells hyperaccumulate intracellular pyridoxal with levels up to 50-fold higher than in healthy cells cultured under an identical pyridoxine-containing medium.[36] These findings are consistent with earlier studies showing that transplantable tumors fail to grow in B6-deficient mice[38,39] and with reports that cancer cells are "addicted" to vitamin B6 due to a dependency on the PLP-dependent enzymes ornithine decarboxylase (ODC1) and glutamic-oxaloacetic transaminase 2 (GOT2).[40] Moreover, vitamin B6 metabolism has been shown to influence antitumoral $CD8^+$ T cell responses,[41] suggesting that cancer cells may suppress immunity by sequestering this essential nutrient from T cells in the tumor microenvironment. We recently found that MR1-restricted cancer-activated T cells could be cultured from the peripheral blood mononuclear cells (PBMCs) of all healthy individuals tested. The majority of these T cells exhibit a semi-invariant TCRα CDR3 motif, analogous to that found in MAIT cells.[5] MR1-restricted, cancer-reactive T cells bearing this TRAJ42-containing motif have been observed expanded and $PD1^+$ among tumor-infiltrating lymphocytes in breast cancer.[42] The principal ligand for MAIT cells, 5-OP-RU, is an adduct of the 5-Amino-6-(D-ribitylamino)uracil (5-A-RU) intermediate in riboflavin (vitamin B2) biosynthesis and methylglyoxal, an antimicrobial agent produced by neutrophils during bacterial infection.[43] Consequently, MAITs recognize a chemical neoantigen generated from two molecules likely to be enriched at sites of microbial infection. By analogy, MR1-restricted recognition of cancer may require a vitamin B6-derived neoantigen. Intriguingly, one such compound, adenosine-N6-diethylthioether-N-pyridoximine-5′-phosphate, has been proposed as a circulating human tumor marker,[44] implying that B6-containing neoantigen adducts can arise in cancer cells. Indeed, one study found that this compound could account for up to 30% of the total intracellular vitamin B6 in tumor cells cultured with radiolabeled pyridoxine,[45,46] suggesting it is not a minor species. Further studies will be required to determine whether MR1-presented B6-related compounds are of relevance to MR1-restricted recognition of cancer cells.

In summary, we describe a pipeline for MR1 ligand discovery. Importantly, this methodology was able to capture ligands bound to MR1 expressed in cells rather than refolded *in vitro*. We identified pyridoxal and PLP as being bound to MR1 and confirmed that these B6 vitamers could induce upregulation of MR1 as detected by flow cytometry with anti-MR1 antibody. We further demonstrated that pyridoxal could act as an MR1-restricted ligand for T cells bearing the A-F7 canonical MAIT TCR or the non-MAIT MC.7.G5 TCR. Further work will be required to determine whether T cell recognition of B6 vitamers or derivatives of these compounds is relevant in the context of disease.

### Limitations of the study

Our approach is limited to ligands that form a Schiff base with the MR1 α chain. While we successfully identified two previously unreported ligands of MR1, pyridoxal and PLP, the current pipeline only reports their accurate molecular mass. In principle, MS/MS spectra could be interrogated for fragment ions to infer structure; however, this is currently hindered by the low abundance of relevant signals, limiting the feasibility of such analyses in practice. To circumvent this challenge, we employed a shortlisting strategy based on molecular mass followed by biological validation. Although this approach does not yet permit a fully automatized antigen detection pipeline such as those established for peptide antigens presented on MHC, it allows for informed candidate selection, as demonstrated in this study. Limitations in sensitivity due to the low expression of MR1, even in overexpression systems, currently limit the workflow to applications in cell culture systems. It is anticipated that future improvements in sensitivity should enable MR1 ligand discovery from more limited sources, such as tissue biopsies or primary cells.

During our proof-of-principle experiments, we relied on commercially sourced diclofenac-loaded MR1 to test the feasibility of ligand exchange and reductive cross-linking for Ac-6-FP-loaded MR1. Due to the high cost of this reagent, we tested a range of reducing agent concentrations in a single experiment to maximize the chances of detecting the Ac-6-FP-modified peptide product. Although we were able to detect the product under all five tested conditions, we were unable to repeat these experiments, assess levels of background amination in the absence of $NaCNBH_3$, or explore additional ligands beyond Ac-6-FP.

## RESOURCE AVAILABILITY

### Lead contact

Further information and requests for resources and reagents should be directed to and will be fulfilled by the lead contact, Nicola Ternette (nternette001@dundee.ac.uk).

### Materials availability

All newly generated materials are available from the lead contact upon reasonable request and subject to a completed materials transfer agreement.

### Data and code availability

- Raw data of LC-MS/MS analysis are available via Passel using dataset identifier PASS05867.
- Data analysis pipeline used for shortlisting candidate ligands based on the presence of reporter ions is available for download at https://doi.org/10.5281/zenodo.15488756.
- Any additional information required to reanalyze the data reported in this work paper is available from the lead contact upon request.

## ACKNOWLEDGMENTS

The authors would like to thank all the members of the Ternette laboratory, the Sewell laboratory, the Schmidlin laboratory, and the employees of Enara Bio Ltd. at the time of the study. Dr. Matthais Eberl (Cardiff University) is acknowledged for providing the *Mycobacterium smegmatis* mc$^2$155 used for the study. T.S. acknowledges funding from Enara Bio Ltd., the Oxford University Health Research Bridging Salary Scheme (HRBBS 0011045), and the German Federal Ministry for Education and Research as part of the DIASyM project under grant number 161L0218. A.K.S. is a Wellcome Investigator (220295/Z/20/Z). The figures were partly generated using BioRender (https://biorender.com). Open access funding was enabled and organized by Projekt DEAL.

## AUTHOR CONTRIBUTIONS

Conceptualization, T.S., H.T., G.D., A.K.S., and N.T.; methodology, T.S., H.T., G.D., F.M., P.E.B., O.B.S., J.D.S., A.K.S., and N.T.; software, T.S. and M.v.E.; validation, all authors; formal analysis, T.S. and H.T.; investigation, T.S., E.B., H.T., G.D., F.M., S.H., M.v.E., R.M.G., and B.A.S.; resources, F.M., J.D., O.B. S., J.D.S., A.K.S., and N.T.; data curation, T.S. and N.T.; writing – original draft, T.S., A.K.S., and N.T.; writing – review & editing, all authors; visualization, T.S., E.B., H.T., and N.T.; supervision, J.D., J.D.S., A.K.S., and N.T.; project administration, T.S., H.S., A.K.S., and N.T.; funding acquisition, T.S., J.D., A.K.S., and N.T.

## DECLARATION OF INTERESTS

F.M., M.v.E., R.M.G., H.S., J.D., J.D.S., and N.T. were employees of Enara Bio at the time of this study and were in receipt of salary and stock options in Enara Bio Ltd. For parts of the study, T.S., E.B., and S.H. were academic postdoctoral researchers fully or partly funded by Enara Bio Ltd. A.K.S. and G.D. have patents granted and pending on T cell recognition.

## STAR★METHODS

Detailed methods are provided in the online version of this paper and include the following:


- KEY RESOURCES TABLE
- EXPERIMENTAL MODEL AND STUDY PARTICIPANT DETAILS
  - HEK293T cell line culture
  - MM909.24 cell line culture
  - C1R cell line culture
  - A549 cell line culture
  - E6.1 jurkat cell line culture
  - Triple parameter reporter (TPR) jurkat cell line culture
  - Primary T cell culture
  - Mycoplasma testing
- METHOD DETAILS
  - *In silico* digestion and optimization of proteolytic enzyme
  - Design of tagged scMR1 constructs
  - Site directed mutagenesis
  - MR1 lentivirus production and transduction of cell lines
  - Flow cytometry and cell sorting of MR1 transduced cells
  - Acetyl-6-formylpterin (Ac-6-FP) pulsing
  - Enzyme linked immunosorbant assays (ELISA)
  - Mycobacterium smegmatis infection
  - TCR lentivirus production and transduction of TPR jurkat cells and primary T cells
  - Cell lysis
  - Enrichment of endogenous MR1 by immunoprecipitation
  - Strep-Tactin enrichment
  - Cross-linking by reductive amination
  - Chymotrypsin digestion
  - Sample desalting and purification
  - LC-MS/MS analysis
  - Synthesis of DSVTRQ**K**(Ac-6-FP)EPRAPW peptide standard
  - *In vitro* MR1 ligand exchange and cross-linking
  - Data analysis–database search
  - Data analysis–shortlisting candidate spectra for DSVTRQ**K**EPRAPW bound to ligands of unknown mass
  - Mass error estimation for Δ-mass values
  - Benchmarking of data analysis pipeline on MM909.24 cells
  - Shortlisting candidate compounds for observed Δ-mass values
  - Testing upregulated MR1 surface expression for selected compounds
  - CD69 assays
  - T107 assay
  - Visualization
- QUANTIFICATION AND STATISTICAL ANALYSIS


## SUPPLEMENTAL INFORMATION

Supplemental information can be found online at https://doi.org/10.1016/j.crmeth.2025.101120.

Received: July 5, 2024
Revised: November 24, 2024
Accepted: July 8, 2025
Published: August 4, 2025

## REFERENCES


1. Kjer-Nielsen, L., Patel, O., Corbett, A.J., Le Nours, J., Meehan, B., Liu, L., Bhati, M., Chen, Z., Kostenko, L., Reantragoon, R., et al. (2012). MR1 presents microbial vitamin B metabolites to MAIT cells. Nature *491*, 717–723. https://doi.org/10.1038/nature11605.
2. Patel, O., Kjer-Nielsen, L., Le Nours, J., Eckle, S.B.G., Birkinshaw, R., Beddoe, T., Corbett, A.J., Liu, L., Miles, J.J., Meehan, B., et al. (2013). Recognition of vitamin B metabolites by mucosal-associated invariant T cells. Nat. Commun. *4*, 2142. https://doi.org/10.1038/ncomms3142.
3. Corbett, A.J., Eckle, S.B.G., Birkinshaw, R.W., Liu, L., Patel, O., Mahony, J., Chen, Z., Reantragoon, R., Meehan, B., Cao, H., et al. (2014). T-cell activation by transitory neo-antigens derived from distinct microbial pathways. Nature *509*, 361–365. https://doi.org/10.1038/nature13160.
4. Crowther, M.D., Dolton, G., Legut, M., Caillaud, M.E., Lloyd, A., Attaf, M., Galloway, S.A.E., Rius, C., Farrell, C.P., Szomolay, B., et al. (2020). Genome-wide CRISPR-Cas9 screening reveals ubiquitous T cell cancer targeting via the monomorphic MHC class I-related protein MR1. Nat. Immunol. *21*, 178–185. https://doi.org/10.1038/s41590-019-0578-8.
5. Dolton, G., Thomas, H., Tan, L.R., Rius Rafael, C., Doetsch, S., Ionescu, G. A., Cardo, L.F., Crowther, M.D., Behiry, E., Morin, T., et al. (2025). MHC-related protein 1-restricted recognition of cancer via a semi-invariant TCR-alpha chain. J. Clin. Investig. *135*, e181895. https://doi.org/10.1172/JCI181895.
6. Mak, J.Y.W., Xu, W., Reid, R.C., Corbett, A.J., Meehan, B.S., Wang, H., Chen, Z., Rossjohn, J., McCluskey, J., Liu, L., and Fairlie, D.P. (2017). Stabilizing short-lived Schiff base derivatives of 5-aminouracils that activate mucosal-associated invariant T cells. Nat. Commun. *8*, 14599.

7. McWilliam, H.E.G., Eckle, S.B.G., Theodossis, A., Liu, L., Chen, Z., Wubben, J.M., Fairlie, D.P., Strugnell, R.A., Mintern, J.D., McCluskey, J., et al. (2016). The intracellular pathway for the presentation of vitamin B-related antigens by the antigen-presenting molecule MR1. Nat. Immunol. *17*, 531–537. https://doi.org/10.1038/ni.3416.

8. Awad, W., Ler, G.J.M., Xu, W., Keller, A.N., Mak, J.Y.W., Lim, X.Y., Liu, L., Eckle, S.B.G., Le Nours, J., McCluskey, J., et al. (2020). The molecular basis underpinning the potency and specificity of MAIT cell antigens. Nat. Immunol. *21*, 400–411. https://doi.org/10.1038/s41590-020-0616-6.

9. Harriff, M.J., McMurtrey, C., Froyd, C.A., Jin, H., Cansler, M., Null, M., Worley, A., Meermeier, E.W., Swarbrick, G., Nilsen, A., et al. (2018). MR1 displays the microbial metabolome driving selective MR1-restricted T cell receptor usage. Sci. Immunol. *3*, eaao2556. https://doi.org/10.1126/sciimmunol.aao2556.

10. Ler, G.J.M., Xu, W., Mak, J.Y.W., Liu, L., Bernhardt, P.V., and Fairlie, D.P. (2019). Computer Modelling and Synthesis of Deoxy and Monohydroxy Analogues of a Ribitylaminouracil Bacterial Metabolite that Potently Activates Human T Cells. Chemistry *25*, 15594–15608. https://doi.org/10.1002/chem.201903732.

11. Salio, M., Awad, W., Veerapen, N., Gonzalez-Lopez, C., Kulicke, C., Waithe, D., Martens, A.W.J., Lewinsohn, D.M., Hobrath, J.V., Cox, L.R., et al. (2020). Ligand-dependent downregulation of MR1 cell surface expression. Proc. Natl. Acad. Sci. USA *117*, 10465–10475. https://doi.org/10.1073/pnas.2003136117.

12. McWilliam, H.E.G., and Villadangos, J.A. (2024). MR1 antigen presentation to MAIT cells and other MR1-restricted T cells. Nat. Rev. Immunol. *24*, 178–192. https://doi.org/10.1038/s41577-023-00934-1.

13. Vacchini, A., Chancellor, A., Yang, Q., Colombo, R., Spagnuolo, J., Berloffa, G., Joss, D., Øyås, O., Lecchi, C., De Simone, G., et al. (2024). Nucleobase adducts bind MR1 and stimulate MR1-restricted T cells. Sci. Immunol. *9*, eadn0126. https://doi.org/10.1126/sciimmunol.adn0126.

14. Krawic, J.R., Ladd, N.A., Cansler, M., McMurtrey, C., Devereaux, J., Worley, A., Ahmed, T., Froyd, C., Kulicke, C.A., Swarbrick, G., et al. (2024). Multiple Isomers of Photolumazine V Bind MR1 and Differentially Activate MAIT Cells. J. Immunol. *212*, 933–940. https://doi.org/10.4049/jimmunol.2300609.

15. Purcell, A.W., Ramarathinam, S.H., and Ternette, N. (2019). Mass spectrometry-based identification of MHC-bound peptides for immunopeptidomics. Nat. Protoc. *14*, 1687–1707. https://doi.org/10.1038/s41596-019-0133-y.

16. Eckle, S.B.G., Birkinshaw, R.W., Kostenko, L., Corbett, A.J., McWilliam, H.E.G., Reantragoon, R., Chen, Z., Gherardin, N.A., Beddoe, T., Liu, L., et al. (2014). A molecular basis underpinning the T cell receptor heterogeneity of mucosal-associated invariant T cells. J. Exp. Med. *211*, 1585–1600. https://doi.org/10.1084/jem.20140484.

17. Boersema, P.J., Raijmakers, R., Lemeer, S., Mohammed, S., and Heck, A.J.R. (2009). Multiplex peptide stable isotope dimethyl labeling for quantitative proteomics. Nat. Protoc. *4*, 484–494. https://doi.org/10.1038/nprot.2009.21.

18. Holden, C.A., Yuan, Q., Yeudall, W.A., Lebman, D.A., and Yang, H. (2010). Surface engineering of macrophages with nanoparticles to generate a cell-nanoparticle hybrid vehicle for hypoxia-targeted drug delivery. Int. J. Nanomedicine *5*, 25–36.

19. Keller, A.N., Eckle, S.B.G., Xu, W., Liu, L., Hughes, V.A., Mak, J.Y.W., Meehan, B.S., Pediongco, T., Birkinshaw, R.W., Chen, Z., et al. (2017). Drugs and drug-like molecules can modulate the function of mucosal-associated invariant T cells. Nat. Immunol. *18*, 402–411. https://doi.org/10.1038/ni.3679.

20. Huang, S., Gilfillan, S., Cella, M., Miley, M.J., Lantz, O., Lybarger, L., Fremont, D.H., and Hansen, T.H. (2005). Evidence for MR1 antigen presentation to mucosal-associated invariant T cells. J. Biol. Chem. *280*, 21183–21193. https://doi.org/10.1074/jbc.M501087200.

21. Reantragoon, R., Kjer-Nielsen, L., Patel, O., Chen, Z., Illing, P.T., Bhati, M., Kostenko, L., Bharadwaj, M., Meehan, B., Hansen, T.H., et al. (2012). Structural insight into MR1-mediated recognition of the mucosal associated invariant T cell receptor. J. Exp. Med. *209*, 761–774. https://doi.org/10.1084/jem.20112095.

22. Laugel, B., Lloyd, A., Meermeier, E.W., Crowther, M.D., Connor, T.R., Dolton, G., Miles, J.J., Burrows, S.R., Gold, M.C., Lewinsohn, D.M., and Sewell, A.K. (2016). Engineering of Isogenic Cells Deficient for MR1 with a CRISPR/Cas9 Lentiviral System: Tools To Study Microbial Antigen Processing and Presentation to Human MR1-Restricted T Cells. J. Immunol. *197*, 971–982. https://doi.org/10.4049/jimmunol.1501402.

23. Kong, A.T., Leprevost, F.V., Avtonomov, D.M., Mellacheruvu, D., and Nesvizhskii, A.I. (2017). MSFragger: ultrafast and comprehensive peptide identification in mass spectrometry-based proteomics. Nat. Methods *14*, 513–520. https://doi.org/10.1038/nmeth.4256.

24. Sinitcyn, P., Gerwien, M., and Cox, J. (2022). MaxQuant Module for the Identification of Genomic Variants Propagated into Peptides. Methods Mol. Biol. *2456*, 339–347. https://doi.org/10.1007/978-1-0716-2124-0_23.

25. Garcia, C.A., Gil-de-la-Fuente, A., Barbas, C., and Otero, A. (2022). Probabilistic metabolite annotation using retention time prediction and meta-learned projections. J Cheminform *14*, 33. https://doi.org/10.1186/s13321-022-00613-8.

26. Dawadi, S., Kordus, S.L., Baughn, A.D., and Aldrich, C.C. (2017). Synthesis and Analysis of Bacterial Folate Metabolism Intermediates and Antifolates. Org. Lett. *19*, 5220–5223. https://doi.org/10.1021/acs.orglett.7b02487.

27. Da Silva, V.R., and Gregory, I.I.I.J.F. (2020). Vitamin B6. In Present Knowledge in Nutrition, B.P. Marriott, D.F. Birt, V.A. Stallings, and A.A. Y, eds. (Academic Press (Elsevier), pp. 225–238.

28. Muller, T.R., Schuler, C., Hammel, M., Kohler, A., Jutz, S., Leitner, J., Schober, K., Busch, D.H., and Steinberger, P. (2020). A T-cell reporter platform for high-throughput and reliable investigation of TCR function and biology. Clin Transl Immunology *9*, e1216. https://doi.org/10.1002/cti2.1216.

29. Tilloy, F., Treiner, E., Park, S.H., Garcia, C., Lemonnier, F., de la Salle, H., Bendelac, A., Bonneville, M., and Lantz, O. (1999). An invariant T cell receptor alpha chain defines a novel TAP-independent major histocompatibility complex class Ib-restricted alpha/beta T cell subpopulation in mammals. J. Exp. Med. *189*, 1907–1921. https://doi.org/10.1084/jem.189.12.1907.

30. Midttun, O., Hustad, S., Solheim, E., Schneede, J., and Ueland, P.M. (2005). Multianalyte quantification of vitamin B6 and B2 species in the nanomolar range in human plasma by liquid chromatography-tandem mass spectrometry. Clin. Chem. *51*, 1206–1216. https://doi.org/10.1373/clinchem.2005.051169.

31. Van den Eynde, M.D.G., Scheijen, J.L.J.M., Stehouwer, C.D.A., Miyata, T., and Schalkwijk, C.G. (2021). Quantification of the B6 vitamers in human plasma and urine in a study with pyridoxamine as an oral supplement; pyridoxamine as an alternative for pyridoxine. Clin. Nutr. *40*, 4624–4632. https://doi.org/10.1016/j.clnu.2021.05.028.

32. Richts, B., Rosenberg, J., and Commichau, F.M. (2019). A Survey of Pyridoxal 5'-Phosphate-Dependent Proteins in the Gram-Positive Model Bacterium Bacillus subtilis. Front. Mol. Biosci. *6*, 32. https://doi.org/10.3389/fmolb.2019.00032.

33. Galluzzi, L., Vacchelli, E., Michels, J., Garcia, P., Kepp, O., Senovilla, L., Vitale, I., and Kroemer, G. (2013). Effects of vitamin B6 metabolism on oncogenesis, tumor progression and therapeutic responses. Oncogene *32*, 4995–5004. https://doi.org/10.1038/onc.2012.623.

34. Galluzzi, L., Vitale, I., Senovilla, L., Olaussen, K.A., Pinna, G., Eisenberg, T., Goubar, A., Martins, I., Michels, J., Kratassiouk, G., et al. (2012). Prognostic impact of vitamin B6 metabolism in lung cancer. Cell Rep. *2*, 257–269. https://doi.org/10.1016/j.celrep.2012.06.017.

35. Gridley, D.S., Stickney, D.R., Nutter, R.L., Slater, J.M., and Shultz, T.D. (1987). Suppression of tumor growth and enhancement of immune status with high levels of dietary vitamin B6 in BALB/c mice. J. Natl. Cancer Inst. *78*, 951–959.

36. McInerney, M.P., Awad, W., Souter, M.N.T., Kang, Y., Wang, C.J.H., Chan Yew Poa, K., Abdelaal, M.R., Le, N.H., Shepherd, C.M., McNeice, C., et al. (2024). MR1 presents vitamin B6-related compounds for recognition by MR1-reactive T cells. Proc. Natl. Acad. Sci. USA *121*, e2414792121. https://doi.org/10.1073/pnas.2414792121.

37. Rozemuller, E., Eckle, S.B.G., McLaughlin, I., Penning, M., Mulder, W., de Bruin, H., and van Wageningen, S. (2021). MR1 encompasses at least six allele groups with coding region alterations. HLA *98*, 509–516. https://doi.org/10.1111/tan.14390.

38. Ferrer, J.F., and Mihich, E. (1967). Dependence of the Regression of Sarcoma 180 in Vitamin B6- deficient Mice upon the Immunologic Competence of the Host. Cancer Res. *27*, 456–461.

39. Fortmeyer, H.P., Timm, C., Blum, U., Wenisch, H.J., and Förster, H. (1988). Vitamin B6 responsive growth of human tumors. Anticancer Res. *8*, 813–818.

40. Chen, C.C., Li, B., Millman, S.E., Chen, C., Li, X., Morris, J.P., 4th, Mayle, A., Ho, Y.J., Loizou, E., Liu, H., et al. (2020). Vitamin B6 Addiction in Acute Myeloid Leukemia. Cancer Cell *37*, 71–84. https://doi.org/10.1016/j.ccell.2019.12.002.

41. Bargiela, D., Cunha, P.P., Veliça, P., Foskolou, I.P., Barbieri, L., Rundqvist, H., and Johnson, R.S. (2022). Vitamin B6 Metabolism Determines T Cell Anti-Tumor Responses. Front. Immunol. *13*, 837669. https://doi.org/10.3389/fimmu.2022.837669.

42. Hayee, A., Kobayashi, E., Motozono, C., Hamana, H., My, H.T.V., Okada, T., Toyooka, N., Yamaguchi, S., Ozawa, T., and Kishi, H. (2024). Characterization of Tumor-Infiltrating Lymphocyte-Derived Atypical TCRs Recognizing Breast Cancer in an MR1-Dependent Manner. Cells *13*, 1711. https://doi.org/10.3390/cells13201711.

43. Zhang, M.M., Ong, C.I.Y., Walker, M.J., and McEwan, A.G. (2016). Defence against methylglyoxal in Group A Streptococcus: a role for Glyoxylase I in bacterial virulence and survival in neutrophils? Pathog. Dis. *74*, ftv122. https://doi.org/10.1093/femspd/ftv122.

44. Tryfiates, G.P., Gannett, P.M., Bishop, R.E., Shastri, P.K., Ammons, J.R., and Arbogast, J.G. (1996). Vitamin B6 and cancer: synthesis and occurrence of adenosine-N6-diethylthioether-N-pyridoximine-5'-phosphate, a circulating human tumor marker. Cancer Res. *56*, 3670–3677.

45. Bishop, R.E., and Tryfiates, G.P. (1989). Biosynthesis of a novel form of vitamin B-6 by tumor cells. J. Nutr. *119*, 189–195. https://doi.org/10.1093/jn/119.2.189.

46. Gregory, J.F. (1992). A novel vitamin B6 metabolite may be a circulating marker of cancer. Nutr. Rev. *50*, 295–297. https://doi.org/10.1111/j.1753-4887.1992.tb02470.x.

47. MacLean, B., Tomazela, D.M., Shulman, N., Chambers, M., Finney, G.L., Frewen, B., Kern, R., Tabb, D.L., Liebler, D.C., and MacCoss, M.J. (2010). Skyline: an open source document editor for creating and analyzing targeted proteomics experiments. Bioinformatics *26*, 966–968. https://doi.org/10.1093/bioinformatics/btq054.

48. Ewels, P., Magnusson, M., Lundin, S., and Käller, M. (2016). MultiQC: summarize analysis results for multiple tools and samples in a single report. Bioinformatics *32*, 3047–3048. https://doi.org/10.1093/bioinformatics/btw354.

49. Kockmann, T., and Panse, C. (2021). The rawrr R Package: Direct Access to Orbitrap Data and Beyond. J. Proteome Res. *20*, 2028–2034. https://doi.org/10.1021/acs.jproteome.0c00866.

50. Garcia, C.J., García-Villalba, R., Garrido, Y., Gil, M.I., and Tomás-Barberán, F.A. (2016). Untargeted metabolomics approach using UPLC-ESI-QTOF-MS to explore the metabolome of fresh-cut iceberg lettuce. Metabolomics *12*, 1–12. https://doi.org/10.1007/s11306-016-1082-x.

51. Andersen, R., Donia, M., Ellebaek, E., Holz Borch, T., Kongsted, P., Zeeberg Iversen, T., Rosenkrantz Hölmich, L., Westergren Hendel, H., Met, Ö., Andersen, M.H., et al. (2016). Long-Lasting Complete Responses in Patients with Metastatic Melanoma after Adoptive Cell Therapy with Tumor-Infiltrating Lymphocytes and an Attenuated IL2 Regimen. Clin. Cancer Res. *22*, 3734–3745.

52. Gherardin, N.A., Keller, A.N., Woolley, R.E., Le Nours, J., Ritchie, D.S., Neeson, P.J., Birkinshaw, R.W., Eckle, S.B.G., Waddington, J.N., Liu, L., et al. (2016). Diversity of T Cells Restricted by the MHC Class I-Related Molecule MR1 Facilitates Differential Antigen Recognition. Immunity *44*, 32–45. https://doi.org/10.1016/j.immuni.2015.12.005.

53. Korndorfer, I.P., and Skerra, A. (2002). Improved affinity of engineered streptavidin for the Strep-tag II peptide is due to a fixed open conformation of the lid-like loop at the binding site. Protein Sci. *11*, 883–893. https://doi.org/10.1110/ps.4150102.

54. Trinh, R., Gurbaxani, B., Morrison, S.L., and Seyfzadeh, M. (2004). Optimization of codon pair use within the (GGGGS)3 linker sequence results in enhanced protein expression. Mol. Immunol. *40*, 717–722. https://doi.org/10.1016/j.molimm.2003.08.006.

## STAR★METHODS

### KEY RESOURCES TABLE

| REAGENT or RESOURCE | SOURCE | IDENTIFIER |
|---|---|---|
| Antibodies | | |
| Anti MR1: clone 26.5 | BioLegend | RRID:AB_2562969 |
| Anti-rCD2: clone OX-34 FITC conjugated | BioLegend | RRID:AB_2228899 |
| Anti-rCD2: clone OX-34 PE conjugated | BioLegend | RRID:AB_2073811 |
| Anti-CD3: clone BW264/56, PerCP conjugated | Miltenyi Biotec | RRID:AB_2725959 |
| CD8 APC Vio770: clone BW135/80 | Miltenyi Biotec | RRID:AB_2725983 |
| CD69 APC: clone FN50 | BioLegend | RRID:AB_2922660 |
| Anti TNF PE Vio770: clone cA2 | Miltenyi Biotec | RRID:AB_2784483 |
| Anti CD107a PE: clone H4A3 | BD Biosciences | RRID:AB_10565964 |
| Anti CD8 REA734, APC conjugated | Miltenyi Biotec | RRID:AB_2659237 |
| Bacterial and virus strains | | |
| XL10-Gold Ultracompetent cells | Agilent | Cat# 200315 |
| *Mycobacterium smegmatis* strain mc2155 | provided by Professor Matthias Eberl, Cardiff University | ATCC Cat# 700084 |
| Third generation lentiviral transfer vector backbone pELNS: XbaI-Kozak-β2M-GS linker-MR1 (without leader sequence)-XhoI-P2A-rCD2-SalI-Stop | provided by Dr. James Riley, University of Pennsylvania, PA, USA | N/A |
| Biological samples | | |
| PBMC from 3 donors (EDTA treated buffy coats) | Welsh Blood Service | N/A |
| Chemicals, peptides, and recombinant proteins | | |
| Carbenicillin disodium salt | ThermoFisher Scientific | Cat# 10396833 |
| Agar bacteriological | Fisher Scientific | Cat# 10048991 |
| Opti-MEM | ThermoFisher Scientific | Cat# 31985070 |
| Polyethylenimine | Merck | Cat# 306185 |
| Dulbecco modified Eagle-Medium (DMEM) | Merck | Cat# D6429 |
| Fetal Bovine Serum (FBS) | Merck | Cat# F7524 |
| Penicillin-Streptomycin | Merck | Cat# P4333 |
| L-glutamine | Merck | Cat# G7513 |
| Hexadimethrine bromide (polybrene®) | Santa Cruz | Cat# sc-134220 |
| Roswell Park Memorial Institute 1640 medium (RPMI 1640) | Merck | Cat# R8759 |
| Dulbecco's Phosphate Buffered Saline (D-PBS) | Merck | Cat# D8537 |
| EDTA | Merck | Cat# 6758 |
| Phytohemagglutinin | ThermoScientific | Cat# 10576015 |
| IL-2 (Aldesleukin, Proleukin; Prometheus) | Sourced from Cardiff hospital pharmacy | Cat# 73776-0022-01 |
| IL-15 | Miltenyi Biotec | Cat# 130-095-760 |
| HEPES | Thermo Scientific | Cat# 15630056 |
| Sodium pyruvate | Thermo Scientific | Cat# 11360070 |
| MEM non-essential amino acids | Thermo Scientific | Cat# 11140050 |
| Acetyl-6-formylpterin | Cayman Chemicals<br>Schircks Laboratories | Item No. 23303<br>Product number 11.418 |

(*Continued on next page*)

***Continued***

| REAGENT or RESOURCE | SOURCE | IDENTIFIER |
|---|---|---|
| DMSO | Thermo Scientific (Invitrogen$^{TM}$) | Cat# D12345 |
| Glycerol | Fisher Scientific | Cat# 327255000 |
| Lemco powder | Fisher Scientific | Cat# 10443833 |
| Tryptone | Sigma-Aldrich | Cat# 61044-5KG |
| Sodium Chloride (NaCl) | Fisher Scientific | Cat# 10194763 |
| Tween-80 | Merck | Cat# P4780 |
| Tris base | Thermo Scientific | Cat# BP152-500 |
| NP-40 | Thermo Scientific | Cat# PI85124 |
| Streptavidin | Thermo Scientific | Cat# PI21122 |
| cOmplete Protease Inhibitor Cocktail EDTA-free | Roche, purchased at Merck | Cat# 11873580001 |
| rProtein A Sepharose Fast Flow antibody purification resin | Cytivia | Cat# 17127904 |
| Potassium chloride (KCL) | Thermo Scientific | Cat# AA1159530 |
| Triethanolamine | Fisher Scientific | Cat# T407-1 |
| Dimethyl pimelimidate dihydrochloride | Sigma | Cat# D8388 |
| Citric acid | Fisher Scientific | Cat# 464541000 |
| Acetic acid | Fisher Scientific | Cat# 219992500 |
| Urea | Fisher Scientific | Cat# U15-3 |
| Chymotrypsin, Sequencing Grade | Promega | Cat# V1061 |
| Triethylammoniumbicaronate buffer (TEAB) | Merck | Cat# T7408 |
| Biotin | Merck | Cat# B4639 |
| PBS | Thermo Scientific | Cat# 10010001 |
| TFA | Thermo Scientific | Cat# 293810250 |
| Methanol | Thermo Scientific | Cat# 047192.K2 |
| Acetonitrile | Thermo Scientific | Cat# 047138.K2 |
| Formic Acid | Thermo Scientific | Cat# 147932500 |
| Custom peptide synthesis: DSVTRQ**K**EPRAPW PepPower$^{TM}$ Fast Peptide Synthesis, purity ≥95% | GenScript | Cat# SC1848 |
| scβ2M/MR1 complex with diclofenac | PEAKS Proteins | Project specific custom product |
| $NaCNBH_3$ | Sigma Aldrich Merck | Cat# 156159-10G |
| Pyridoxal hydrochloride | Sigma Aldrich | Cat# P9130 |
| Pyridoxal 5′-phosphate | Fluorochem | Cat# F044568 |
| anti-PE microbeads | Miltenyi Biotec | Cat# 130-048-801 |
| 5-Amino-6-(D-ribitylamino)uracil (5-A-RU) | TargetMol | Cat# T10165 |
| CD3/CD28 Dynabeads | ThermoFischer | Cat# 40203D |
| Phorbol 12-Myristate 13-Acetate (PMA) | Promega | Cat# V1171 |
| anti-CD8 microbeads | Miltenyi Biotec | Cat# 130-045-201 |
| TNF-processing inhibitor (TAPI)-0 | Merck | Cat# 579050 |
| **Critical commercial assays** | | |
| Prime star PCR Master Mix | Takara | Cat# R053A |
| Kinase-Ligase-Dpn1 enzyme mix | New England Biolabs | Cat# M0554S |
| HiPure Miniprep kit | ThermoFisher Scientific | Cat# K210002 |
| MycoAlertTM kit | Lonza | Cat# LT07-318 |
| LIVE/DEAD® Fixable Violet Dead Stain Kit | ThermoScientific | Cat# L34964 |
| FcR block | Miltenyi Biotec | Cat# 130-059-901 |
| TNF DuoSet ELISA kits | R&D Systems | Cat# DY210 |

*(Continued on next page)*

| *Continued* | | |
|---|---|---|
| REAGENT or RESOURCE | SOURCE | IDENTIFIER |
| QIAamp DNA Mini Kit | Qiagen | Cat# 56304 |
| Nextera XT V2 kit | Illumina | Cat# FC-131-2004 |
| 5kDa MWCO filters | Merck | Cat# MPE005025 |
| Oasis PRiME HLB 3cc Vac Cartridge | Waters | Cat# WAT094226 |
| LentiBOOST | Sirion Biotech | https://www.pharmaceutical-technology.com/products/lentiboost-products/ |
| Deposited data | | |
| Mass spectrometry raw files | This paper | PASS05867 |
| Experimental models: Cell lines | | |
| HEK293T | ATCC | Cat# CRL-1573 |
| Melanoma line MM909.24 | derived from metastatic lesions at the CCIT-DK | N/A |
| C1R | ATCC | Cat# CRL-1993 |
| C1R.MR1 | This paper | N/A |
| Jurkat E6.1 | ATCC | Cat# TIB-152 |
| A549 | ATCC | Cat# CCL-185 |
| MC.7.G5 | Crowther 2020[4] | N/A |
| Jurkat TCR KO CD8αβ+ triple parameter reporter (TPR) cells | Provided by Professor Peter Steinberger, Vienna, Austria | N/A |
| Oligonucleotides | | |
| Strep-II forward oligo: 5′ CCGACAGA TGGTCTCACCCCCAATTTGAAAAA CTCGAGAGCGGCTCCGGCGAG 3′ | Eurofins Genomics | N/A |
| Strep-II reverse oligo: 5′ CTCTCGAG TTTTTCAAATTGGGGGTGAGACCA TCTGTCGGGGGTGGGCAGG 3′ | Eurofins Genomics | N/A |
| K43A MR1 forward: 5′ GCCGAGCCC AGAGCCCCCTGG 3′ | Eurofins Genomics | N/A |
| K43A MR1 reverse: 5′ GGCCTGTC TGGTCACGCTGTCG 3′ | Eurofins Genomics | N/A |
| pELNS Forward: 5′ GAGTTT GGATCTTGGTTCATTC 3′ | Eurofins Genomics | N/A |
| rat (r) CD2 Reverse: 5′ AACTTGC ACCGCATATGCAT 3′ | Eurofins Genomics | N/A |
| Recombinant DNA | | |
| Single chain (sc) codon-optimized gene for MR1 without its signal sequence (Uniprot.org, Nov. 2022) fused to β2M via a GGGGSGGGGSGGGGS linker | GeneArt | N/A |
| Codon-optimized A-F7 TCR was expressed from the pSF plasmid, with the TRA and TRB chains separated by a self-cleaving T2A, and a P2A between the TRB gene and rCD2 co-marker | This paper | N/A |
| Software and algorithms | | |
| Skyline | MacLean et al. 2010[47] | https://skyline.ms/project/home/begin.view |
| FlowJo | Tree Star Inc. | https://www.flowjo.com/solutions/flowjo |
| Trimgalore v0.6.4 | Babraham Bioinformatics | https://github.com/FelixKrueger/TrimGalore |
| Shovill pipeline | github | https://github.com/tseemann/shovill |

(*Continued on next page*)

*Continued*

| REAGENT or RESOURCE | SOURCE | IDENTIFIER |
|---|---|---|
| fastqc v0.11.8 | Babraham Bioinformatics | https://www.bioinformatics.babraham.ac.uk/projects/fastqc/ |
| BlastN | National Library of Medicine | https://blast.ncbi.nlm.nih.gov/Blast.cgi?PROGRAM=blastn&BLAST_SPEC=GeoBlast&PAGE_TYPE=BlastSearch |
| MultiQC (v1.12) | Ewels et al. 2016[48] | https://github.com/MultiQC/MultiQC |
| Geneious Prime v2022.1.1 | Geneious | https://www.geneious.com/ |
| Peaks v10.0 | Bioinformatics Solutions | https://www.bioinfor.com/ |
| MSconvert | ProteoWizard | https://proteowizard.sourceforge.io/download.html |
| rawrr | Kockmann et al. 2021[49] | https://bioconductor.org/packages/3.21/bioc/html/rawrr.html |
| In house script Δ-mass | This paper | https://doi.org/10.5281/zenodo.15488756 |
| CEU Mass Mediator | Garcia et al. 2016[50] | https://ceumass.eps.uspceu.es/ |
| Other | | |
| BD FACSCanto II | BD Biosciences | https://www.bdbiosciences.com/en-se/products/instruments/flow-cytometers/clinical-cell-analyzers/facscanto |
| ACEA NovoCyte 3005 with NovoSampler pro | ACEA, Agilent | Legacy instrument |
| QIAcube automated extractor | Qiagen | https://www.qiagen.com/us/products/discovery-and-translational-research/dna-rna-purification/instruments-equipment/qiacube-connect |
| Qubit Fluorometer 3.0 | Invitrogen | Cat# Q33216 |
| MiSeq | Illumina | https://www.illumina.com/systems/sequencing-platforms/miseq.html |
| Strep-Tactin XT 4flow column | IBA Lifesciences | https://www.iba-lifesciences.com/strep-tag |
| ÄKTA Pure protein purification system | Cytiva | https://www.cytivalifesciences.com/en/us/shop/chromatography/chromatography-systems/akta-pure-p-05844?srsltid=AfmBOoqR6S7Wc8wa8GLqsh6Zrcjraya-ZnXBvSWJekO4cZ83TL6Pryrw |
| Q Exactive HF-X mass spectrometer | Thermo Scientific | Legacy instrument |
| Fusion Lumos mass spectrometer | Thermo Scientific | Cat# FETD2-10002 |
| Ultimate 3000 RSLCnano System | Thermo Scientific | Cat# ULTIM3000RSLCNANO |
| Acclaim PepMap 100 C18 5 μM 0.1 × 20 mm column | Thermo Scientific | Cat# 164946 |
| 75 μm × 50 cm PepMap RSLC C18 EasySpray | Thermo Scientific | Cat# ES75500PN |
| DynaMag-15 | Thermo Scientific | Cat# 12301D |

## EXPERIMENTAL MODEL AND STUDY PARTICIPANT DETAILS

### HEK293T cell line culture

Human embryo kidney (HEK)293T cell line (ATCC information, CRL-1573) was sourced locally. Cells were cultured and maintained in D10 (Dulbecco Modified Eagle-Medium (Merck, Cat# D6429), 10% FBS (Merck, Cat# F7524), 100U/ml penicillin and 100 μg/mL streptomycin (Merck, Cat# P4333), and 2 mM L-Glutamine (Merck, Cat# G7513)) at 37°C, 5% $CO_2$ in a humidified atmosphere. Cell lines were maintained as monolayers and passaged when they reached 50–80% confluency, using Dulbecco's Phosphate Buffered Saline (Merck, Cat# D8537) and 2mM EDTA (Merck, Cat# 6758) to facilitate cell detachment.

### MM909.24 cell line culture

The melanoma lines from patient MM909.24 were derived from metastatic lesions at the CCIT-DK.[51] Cells were cultured and maintained in R10 (RPMI-1640 (Merck, Cat# R8759), 10% FBS, 100U/ml penicillin and 100 μg/mL streptomycin) at 37°C, 5% $CO_2$ in a humidified atmosphere. Cell lines were maintained as monolayers and passaged when they reached 50–80% confluency, using RPMI-1640 and 2mM EDTA to facilitate cell detachment.

### C1R cell line culture

B-cell lymphoblastoid cell line C1R (ATCC information, CRL-1993) were sourced locally and engineered to overexpress MR1*01 using lentivirus.[52] C1R cell lines were cultured in R10[4] at 37°C, 5% $CO_2$ in a humidified atmosphere.

### A549 cell line culture

Lung adenocarcinoma A549 cells were sourced locally (ATCC information, CCL-185). Cells were cultured in R10 at 37°C, 5% $CO_2$ in a humidified atmosphere. An MR1 knockout A549 cell clone (‘clone 9’) was used for the study and generated as previously described.[22]

### E6.1 jurkat cell line culture

Leukemia E6.1 Jurkat cell line was obtained from ATCC (TIB-152) and cultured in R10 at 37°C, 5% $CO_2$ in a humidified atmosphere.

### Triple parameter reporter (TPR) jurkat cell line culture

Leukemia Jurkat TCR KO CD8αβ+ triple parameter reporter (TPR) cells were provided by Professor Peter Steinberger (Vienna, Austria) and were used for A-F7 and MC.7.G5 TCR expression[28] (see below for lentiviral expression of TCRs). Cells were cultured in R10 at 37°C, 5% $CO_2$ in a humidified atmosphere.

### Primary T cell culture

MR1 restricted clone MC.7.G5 was generated and characterized as previously described.[4] MC.7.G5, MAIT A-F7 and MC.7.G5 TCR-T cells (see below for details of TCR-T cells) were expanded with irradiated (3000–3100 cGy) peripheral mononuclear cells (PBMCs) from three donors, sourced from the Welsh Blood Service (Pontyclun, Wales, UK) as EDTA treated ‘buffy coats’ (ethical approval granted by the Cardiff University School of Medicine Research Ethics Committee, ref. 18/56), with 1 μg/mL of phytohemagglutinin (PHA) (Remel, Thermo Scientific). Media comprised R10 (as above) supplemented with 20 IU/mL of IL-2 (Aldesleukin, Proleukin, Prometheus), 25 ng/mL of IL-15 (Miltenyi Biotec), 10 mM HEPES, 1 mM sodium pyruvate and 1X MEM non-essential amino acids (Thermo Scientific, unless indicated otherwise). For expansion, 0.1–0.2 $\times$ $10^6$ T cells and $4\times10^6$ irradiated PBMCs were plated per well of a 24 well plate in 2 mL of media. After 5 days 50% of the media was changed and on day 7 the IL-2 concentration was increased to 200 IU/mL. T cells were maintained at 3-$4\times10^6$ per well on a 24 well plate and 50% of the media changed thrice weekly (see table Summary of cell culture medium used for various cell lines).

Summary of cell culture medium used for various cell lines.

| Cell line | Culture Medium |
|---|---|
| HEK293T | D10 |
| MM909.24 | R10 |
| C1R | R10 |
| A549 | R10 |
| Jurkat | R10 |
| Primary T cells | R10 |

### Mycoplasma testing

All cells were subjected to regular mycoplasma testing using the MycoAlert kit (Lonza, Catalog #: LT07-318).

## METHOD DETAILS

### *In silico* digestion and optimization of proteolytic enzyme

*In silico* digests for the canonical MR1 sequence (Uniprot.org, Nov. 2022) were performed with various proteolytic enzymes using Skyline.[47]

### Design of tagged scMR1 constructs

A single chain (sc) codon-optimized gene for MR1 without its signal sequence (Uniprot.org, Nov. 2022) fused to β2M via a GGGGSGGGGSGGGGS linker, was synthesized by GeneArt (Life Technologies). A strep-II tag[53] was added to the scβ2M/MR1

(scMR1) construct by site directed mutagenesis using the following oligonucleotides: Strep-II forward oligo (5′ CCGACAGATGGTCT CACCCCCAATTTGAAAAACTCGAGAGCGGCTCCGGCGAG 3′) and Strep-II reverse oligo (5′ CTCTCGAGTTTTTCAAATTGGGGGT GAGACCATCTGTCGGGGGTGGGCAGG 3′), inserted nucleotides are underlined. A K43A substitution was then added by site directed mutagenesis using the following oligonucleotides: K43A MR1 forward (5′ GCCGAGCCCAGAGCCCCCTGG 3′) and K43A MR1 reverse (5′ GGCCTGTCTGGTCACGCTGTCG 3′), altered nucleotides are underlined. Oligonucleotides were purchased via the Eurofins Genomics Website (Luxembourg, EU).

## Site directed mutagenesis

PCR was performed using Prime star PCR Master Mix (Takara). Cycling conditions were performed according to the manufacturer's instructions, except elongation which was done at 1 min/kb. The PCR products' sizes were verified by running samples on a 1% agarose gel. Correctly amplified products were treated by Kinase-Ligase-Dpn1 enzyme mix (New England Biolabs, Ipswich, MA, USA) for 30 min at room temperature. Samples were then transformed into XL10-Gold Ultracompetent cells (Agilent Technologies Santa Clara, CA, US) by heat shock and cells were plated on LB-Agar plate with Carbenicillin antibiotic (50 μg/mL; ThermoFisher Scientific). Plasmid DNA was extracted from antibiotic resistant colonies using a HiPure Miniprep kit (ThermoFisher Scientific). DNA samples were sent to Eurofins Genomics sequencing platform using the following primers: pELNS Forward: 5′ GAGTTTGGATCT TGGTTCATTC 3′ and rat (r) CD2 Reverse: 5′ AACTTGCACCGCATATGCAT 3'.

## MR1 lentivirus production and transduction of cell lines

The scMR1 strep-II construct was inserted into the third-generation lentiviral transfer vector backbone pELNS (generously provided by Dr. James Riley, University of Pennsylvania, PA, USA). This vector incorporates a rCD2 co-marker gene, preceded by a P2A self-cleaving sequence, in the following order: XbaI-Kozak-β2M-GS linker-MR1 (without leader sequence)-XhoI-P2A-rCD2-SalI-Stop.[54] For transfection, 1.52μg of pELNS (expressing MR1), 0.72μg of envelope plasmid (pMD2.G), and 1.83μg each of packaging plasmids (pMDLg/pRRE and pRSV-REV) were combined in 300 μL of Opti-MEM, a reduced-serum medium from ThermoFisher Scientific. This mixture was then blended with 1 μg/μL Polyethylenimine (PEI; from Merck Group) at a 3:1 PEI to plasmid ratio. The plasmid/PEI amalgamations were allowed to incubate at room temperature for 15 min, after which they were gently dripped onto HEK293T cells cultured in one well of a six-well plate. Supernatant containing lentivirus were collected at 48 h post transfection and stored at 4°C. The media was replenished (3 mL of D10) after the 48 h harvest, then collected at 72 h post transfection. The 48 h and 72 h supernatants were combined and centrifuged at 800 x *g* for 5 min to help remove any HEK293T cells then filtered through a 0.4 μm filter. Lentiviral supernatants were either directly employed for transduction or preserved at −80°C, being defrosted (on ice) only once prior to transduction. In preparation for transduction, 0.1–0.2 x$10^6$ cancer cells were plated 24 h in advance in a well of a 24-well plate with 2 mL of their preferred media. Immediately prior to transduction, the media was aspirated and replaced with 0.5–1 mL of fresh media and lentiviral supernatant added at a 1:1 v/v ratio. Spinfection was performed at 400 x *g* for 2 h in the presence of 5 μg/mL of polybrene (Santa Cruz). Following this, the cells were left to incubate at 37°C overnight, and the media was replaced the subsequent morning. Confirmation of MR1 transduction was carried out 7 days later by staining with MR1 antibody (clone 26.5, BioLegend) and anti-rCD2 antibody (clone OX-34, BioLegend).

## Flow cytometry and cell sorting of MR1 transduced cells

Staining was performed in 5 mL polystyrene test tubes (1 wash per step) or 96U well plates (3 washes per step), using 0.5-1x$10^5$ cells per sample. Dead cells were detected with 2 μL of a 1:40 dilution in PBS of LIVE/DEAD Fixable Violet Dead Stain Kit (ThermoFisher Scientific), stained at room temperature for 5 min, FcR block (Miltenyi Biotec) for 10 min at RT and without washing surface antibodies added then incubated for 20 min on ice. Surface antibodies: MR1 PE or APC (clone 26.5, BioLegend). Samples were acquired on a BD FACSCanto II (BD Biosciences, Franklin Lakes, NJ, USA) or ACEA NovoCyte 3005 with NovoSampler pro (ACEA Biosciences, Agilent, CA, US), then analyzed with FlowJo software (Tree Star Inc., Ashland, Oregon, US). Cells were gated on FSC-A versus SSC-A, single cells (FSC-A versus FSC-H), then viable cells (marker of choice versus LIVE/DEAD Fixable Violet Dead Stain low/negative). Cell sorting was performed on a BD FACS Aria III (BD Biosciences) by Central Biotechnology Services at Cardiff University (Figure S5).

## Acetyl-6-formylpterin (Ac-6-FP) pulsing

Acetyl-6-formylpterin (Ac-6-FP) was purchased from Cayman chemicals and Schircks Laboratories and was reconstituted in DMSO at 50 mg/mL then stored at −20°C protected from light. For MR1 loading, 50–100 μM of Ac-6-FP was incubated overnight at 37°C and 5% $CO_2$ with target cells in their respective media.

## Enzyme linked immunosorbant assays (ELISA)

Prior to activation assays, T cells were 'rested' overnight in R5 medium (R10 but with 5% FBS serum) to help reduce spontaneous release of chemokines and cytokines. $3 \times 10^4$ T cells were used in 96U well plates at 1:2 ratio with antigen-presenting cells or target cells in 100 μL R5 medium. T cells incubated alone or with PHA (10 μg/mL) were used as negative and positive controls respectively. Supernatants (50 μL) were harvested, diluted with 70 μL of R0 (as for R10 but with no serum), then 50 μL used with half-area ELISA plates (Greiner) and TNF DuoSet ELISA kits (R&D Systems, Abingdon, UK), according to the manufacturer's instructions. All assays were performed in duplicate.

### Mycobacterium smegmatis infection

The phagocytic A549 cell line was loaded with *Mycobacterium smegmatis* (strain mc2155, kindly provided by Professor Matthias Eberl, Cardiff University, available at ATCC catalog 700084) as previously described.[22] Confirmation of the *M. smegmatis* strain was validated by whole genome sequencing. A single bacterial colony was transferred into 1.8 mL Lemco broth (Oxoid) and incubated at 37°C and 180 rpm for 18 h. Genomic DNA was extracted using a QIAamp DNA Mini Kit (Qiagen), with an additional RNAse step, on a QIAcube automated extractor (Qiagen), and quantified using a Qubit Fluorometer 3.0. Genomic libraries were prepared using a Nextera XT V2 kit (Illumina) with bead-based normalization. Paired-end WGS was performed on an Illumina MiSeq using the V3 chemistry to generate fragment lengths of up to 300 base pairs (600 cycles). Trimgalore (v0.6.4) was used to remove the Nextera adapter sequences and low-quality bases (–paired –phred33 -q 25 –illumina -e 0.2) and paired-end reads were assembled using the Shovill pipeline showing a depth of coverage >15. Reports were generated using fastqc (v0.11.8) and collated using MultiQC (v1.12). Sequences were analyzed using Geneious Prime (v2022.1.1) and BlastN analysis identified near identical homology for a majority of contigs relative to *M. smegmatis* mc2155 sequences available in NCBI database (accession numbers CP009494, CP001663, and CP000480). For *M. smegmatis* culture, glycerol stock derived bacteria were inoculated into Lemco medium (5 g/L Lemco powder, 10 g/L Tryptone, 5 g/L NaCl (all from Fisher Scientific) and 0.25% Tween-80 (Merck)) and grown for 72 h at 37°C and 170 rpm. Prior to infection of A549 cells, *M. smegmatis* cultures were harvested and centrifuged at 2740 x *g* for 20 min, then washed with excess antibiotic free R10 at 2740 x *g* for 20 min. In preparation for infection, 3.5 x$10^6$ A549 cells (MR1 knockout with scβ2M/MR1) were cultured for 3 days in a T175 flask in R10, then the medium was removed and replaced with antibiotic-free R10 overnight. On the morning of infection the medium was removed from the A549 cultures and replaced with fresh antibiotic free R10 (20 mL), then *M. smegmatis* added to A549 cells at a multiplicity of infection of 100–300:1 (bacteria to A549 cells) based on retrospective colony forming units (CFU) from Lemco agar plates (5 g/L Lemco powder, 10 g/L Tryptone, 5 g/L NaCl and 15 g/L agar, Fisher Scientific) for 2 h in antibiotic-free R10, followed by 2 h incubation with R10 containing 100 U/mL penicillin and 100 μg/mL streptomycin (Life Technologies). A549 cells were washed to remove extracellular *M. smegmatis*. Control uninfected cells (A549 MR1 knockout with scβ2M/MR1) were mock treated as if they had been coincubated with bacteria.

### TCR lentivirus production and transduction of TPR jurkat cells and primary T cells

Codon-optimized A-F7 or MC.7.G5 TCRs were expressed from the pSF plasmid, with the TRA and TRB chains separated by a self-cleaving T2A, and a P2A between the TRB gene and rCD2 co-marker: XbaI-Kozak-TRA-T2A-TRB-XhoI-P2A-rCD2-SalI-Stop. For TPR Jurkat cells, 0.1–0.2 x$10^6$ cells were plated in 1 mL of R10 media with 1 mL of lentiviral supernatant (not concentrated), followed by spinfection at 800 x g for 1.5 h and RT with 0.5 mg/mL of LentiBOOST (Sirion Biotech, Cambridge, MA, USA). The cells were incubated at 37°C overnight and media replaced the following morning. TCR transgene expression in TPR Jurkat cells was confirmed using antibodies for detection of surface expression of CD3 (clone BW264/56, PerCP conjugated, Miltenyi Biotec). The rCD2 co-marker was used for magnetic based enrichments using PE (clone OX34, BioLegend) conjugated rCD2 antibody and anti-PE microbeads (Miltenyi Biotec) following the manufacturer's instructions. For primary T cells, lentiviral production was performed in T175 flasks, using pSF (30 μg), pMD2.G (7.5 μg), pMDLg/pRRE: (15 μg) and pRSV-REV (15 μg), at a 1:3 μg ratio with PEIpro and made up to 750 μL with Opti-MEM. The plasmid/PEIpro was allowed to incubate at RT for 15 min, after which they were added dropwise to the HEK293T cells. Day 2: media was changed for 20 mL of D10. Day 3: supernatant harvested and stored at 4°C. Day 4: supernatant harvested and combined with the day 3 harvest, centrifuged at 800 x g for 5 min and filtered through a 0.4 μm filter, then concentrated using an Optima XPN-80 Ultracentrifuge (Beckman Coulter, Brea, CA) and SW28 rotor, at 4°C and 141,000 x g for 2 h. Supernatants were discarded and pellets resuspended in 0.2 mL of T cell medium containing 200IU/mL of IL-2 and 25 ng/mL of IL-15 and used fresh for transduction without freezing. For transduction of primary T cells, CD8 T cells were purified from healthy donors using anti-CD8 microbeads (Miltenyi Biotec) according to the manufacturer's guidelines, followed by overnight incubation with CD3/CD28 Dynabeads (Life Technologies) at 3:1 bead:T cell ratio in T cell medium (as above). Per transduction, 1x$10^6$ CD8 T cells were used in 1 well of a 48 well plate, with concentrated lentivirus from one T175 flask. On the day of transduction, the medium was removed from the T cells, replaced with 0.5 mL of T cell medium and 0.2 mL of concentrated lentivirus added, then spinfected at 800 x *g* for 1.5 h and RT with 1 mg/mL of LentiBOOST (Sirion Biotech). The CD3/CD28 Dynabeads were removed on day 7 using a DynaMag-15 (Life Technologies). Transduced T cells were enriched on day 10 with anti-rCD2 PE antibody (clone OX34, BioLegend) and anti-PE microbeads according to the manufacturer's instructions (Miltenyi Biotec). Between day 14 and 18 T cells were expanded, as described above.

### Cell lysis

Lysis buffer (5 mM NaCl, 50 mM Tris base with a pH of 7.4, and 0.5% NP-40) was freshly prepared on the day of purification and kept on ice. Approximately $10^9$ cells were washed twice with 50 mL of cold PBS buffer, followed by centrifugation at 400*g* for 5 min. Washed cells were re-suspended in 10–15 mL lysis buffer, supplemented with 0.03 mg of streptavidin and 1x protease inhibitor cocktail. The cell lysate was incubated on ice for 1 h followed by centrifugation at 400 x *g* for 20 min at 4°C to precipitate cellular debris. The resulting supernatant was carefully transferred to a clean centrifuge tube and subjected to centrifugation at 12,000 x *g* for 1 h at 4°C. After a filtration step the supernatant was subjected to Strep-Tactin® enrichment.

### Enrichment of endogenous MR1 by immunoprecipitation

1 mL rProtein A Sepharose Fast Flow antibody purification resin (Cytivia) was washed in 50 mM borate, 50 mM KCl (pH 8.0) solution and then incubated with 2 mg of MR1 antibody clone 26.5 slowly rotating for 1 h at 4°C. The beads were washed in a column format with 0.2 M triethanolamine (pH 8.2), and the bound antibody was cross-linked by incubation with 40 mM dimethyl pimelimidate dihydrochloride (DMP) (Sigma) (pH 8.3) for 1 h at room temperature. Ice-cold 0.2 M Tris buffer (pH 8.0) was added to the mixture to stop the reaction. Unbound antibody was washed off the column with 0.1 M citrate (pH = 3.0), and the column was equilibrated in 50 mM Tris (pH 8.0) for further use. In parallel, 1 mL rProtein A Sepharose Fast Flow antibody purification resin were washed with 50 mM borate, 50 mM KCl (pH 8.0) solution for preclear. Cell lysates from $5x10^8$ Jurkat cells were passed through antibody free beads and flow through was collected for MR1 enrichment. 1 mL 26.5 antibody cross-linked to rProtein A Sepharose Fast Flow antibody purification resin was added to cleared lysates for 1 h, and beads were washed three times with with 50 mM Tris buffer (pH 8.0) containing 150 mM NaCl, then 450 mM NaCl, and finally buffer without NaCl. Bound proteins were then eluted by using 5 mL 10% acetic acid and dried. Samples were resuspended in 8 M urea buffer and sonicated for 15 min and subsequently diluted to 0.5 M urea with 1xPBS prior to chymotryptic digestion and LC-MS/MS analysis. During method development, protocols were tested where the following steps were altered: (1) cell lysates were not passed through antibody-free beads before incubation with the antibody resin, (2) both washing steps including NaCl were omitted and (3) sample were resuspended in 1xPBS instead of 0.5M urea. Samples using this protocol are indicated as “no preclear” (Table S1).

### Strep-Tactin enrichment

scMR1 enrichment was performed using a 1 mL Strep-Tactin XT 4flow column (IBA Lifesciences) coupled to an ÄKTA Pure protein purification system (Cytiva). The column was primed with 4 column volumes (CV) of $ddH_2O$, followed by equilibration with 10 mM TEAB buffer. The filtered supernatant was subsequently loaded onto the column at a flow rate of 1 mL/min. Post-loading, the column was washed with a minimum of 4–6 CV of TEAB buffer. The elution of bound MR1 was accomplished using 4–6 CV of TEAB buffer with 50 mM biotin. Fractions of 1 mL each were collected. 5kDa MWCO filters were used to remove excess biotin prior to reductive cross-linking.

### Cross-linking by reductive amination

Samples obtained from recombinant MR1 or MR1 purified from various cell lysates were incubated with indicated concentrations of $NaCNBH_3$ ranging from 0.1–50 mM. For each concentration point a 25x stock of $NaCNBH_3$ in water was prepared and added at the corresponding concentration. Unless otherwise specified, samples were subsequently incubated at room temperature, 50 rpm shaking for 2 h. 5kDa MWCO filters were used to remove excess reducing agent and buffer exchange to 1X PBS prior to proteolytic digestion.

### Chymotrypsin digestion

Chymotrypsin was purchased from Promega and resuspended in water at a concentration of 0.5 μg/μL. 200 nmol chymotrypsin was added to the total protein amount in the sample followed by incubation at room temperature and light shaking (50 rpm) for 16 h.

### Sample desalting and purification

Peptides were purified using the Oasis PRiME HLB 3cc Vac Cartridge (Waters) according to manufacturer instruction. In brief, samples were acidified with TFA to reach a pH range of 2–2.5 followed by centrifugation at 20,000 g for 10 min. PRiME HLB cartridges were activated once by passing through 1 mL MeOH, equilibrated twice by passing through 0.1%TFA/2%ACN in water. Subsequently, samples were loaded followed by washing twice with 0.1%TFA/2%ACN in water and elution in 1mL 0.1%FA/80%ACN in water. Samples were dried in vacuo and stored at −80°C and resuspended in 0.1%TFA/2%ACN prior to LC-MS/MS analysis.

### LC-MS/MS analysis

Digested samples were dissolved in loading solvent (0.1% (v/v) TFA, 1% (v/v) Acetonitrile) and analyzed by a Q Exactive HF-X mass spectrometer or a Fusion Lumos mass spectrometer coupled to an Ultimate 3000 RSLCnano System (Thermo Scientific). 1% (v/v) DMSO and 0.1% (v/v) formic acid in water was used as buffer A, 1% (v/v) DMSO, 80% (v/v) acetonitrile and 0.1% (v/v) formic acid in water were used as buffer B. Initially, peptides were trapped on an Acclaim PepMap 100 C18 5 μM 0.1 × 20 mm column in loading solvent before analytical separation by a 60 min linear gradient of 2% B to 35% B at a flow rate of 250nL/min on a 75 μm × 50 cm PepMap RSLC C18 EasySpray column at 40°C (Thermo Scientific). An EasySpray source was used to ionize peptides at 2kV with an ion transfer capillary temperature of 305°C. Detection on the Q Exactive HF-X was performed in DDA mode with an MS1 resolution of 60,000 for full MS (320–1600m/z scan range). Top 12 precursors with a minimum intensity of 850 were selected from charge states 2–4 followed by dynamic exclusion of 30s. Selected precursors were isolated using a quadrupole isolation width of 1.3 amu, followed by HCD fragmentation at an nCE of 25. MS2 spectra were measured from 200 to 2000m/z using a resolution of 60,000. Detection on the Orbitrap Lumos was performed in DDA mode with an MS1 resolution of 60,000 for full MS (400–1500m/z scan range). Precursors were selected from charge states 2–5 using top speed mode followed by dynamic exclusion of 60 s. Selected precursors were isolated using a quadrupole isolation width of 1.6 amu, followed by HCD fragmentation at an nCE of 28. MS2 spectra were measured in the Orbitrap detector using automatic scan range mode at a resolution of 15,000.

### Synthesis of DSVTRQK(Ac-6-FP)EPRAPW peptide standard

Custom made synthetic version of DSVTRQ**K**EPRAPW was purchased from GenScript (UK). The dried peptide powder was dissolved in 100 mM TEAB buffer as a 0.625 mM stock solution. Ac-6-FP was dissolved in 100 mM TEAB buffer as an 0.625 mM stock solution and mixed equimolarly at a volume of 100uL. $NaCNBH_3$ was prepared as 25x stock for each of the concentrations 0.1 mM, 1 mM, 24 mM, and 50 mM and added to the reaction in a volume of 4 μL. Reactions occurred at room temperature on a shaker at 50 rpm for 5 min, 10 min, 30 min, 1 h, 2 h, and 16 h respectively. Each reaction condition was performed in triplicate. Reactions were subsequently acidified with TFA to reach pH = 2 and purified with OASIS HLB prior to LC-MS/MS analysis as described above.

### *In vitro* MR1 ligand exchange and cross-linking

A recombinant scβ2M/MR1 complex with a diclofenac ligand was procured from PEAKS proteins. Ligand exchange to Ac-6-FP was induced by incubating 10 μg of scβ2M/MR1 with a 500-fold excess of Ac-6-FP for 16 h at 25°C and 37°C, respectively. A 5kDa MWCO filter was subsequently used to remove excess free ligand and buffer exchange to 100mM TEAB. Samples were subsequently split in 5 equal samples and reduction was performed by adding 0.1 mM $NaCNBH_3$, 1 mM $NaCNBH_3$, 5 mM $NaCNBH_3$, 10 mM $NaCNBH_3$, and 50 mM $NaCNBH_3$ respectively followed by incubation at room temperature, 50rpm shaking for 2h. 5kDa MWCO filters were used to remove excess reducing agent and buffer exchange to 1xPBS. Samples were subsequently digested by chymotrypsin, purified by OASIS HLB and analyzed by LC-MS/MS as described above.

### Data analysis–database search

MS data were analyzed with Peaks v10.0 (Bioinformatics Solutions) for the identification of peptide sequences matching to reviewed SwissProt human protein entries (version download January, 2020). A custom made Ac-6-FP modification was defined according to the expected cross-linking chemistry and used as a variable modification. Remaining search parameters were set as follows: enzyme specificity set to chymotrypsin, peptide tolerance: ±5 ppm, and fragment tolerance: ±0.03 Da. The results were filtered using a peptide-level false discovery rate (FDR) of 1% established through parallel decoy database searches. No PEAKS PTM and SPIDER searches were used. Reported protein quantifications were used for protein abundance plots and assessment of enrichment efficiencies. Skyline was used to visualize XICs of Ac-6-FP bound and unbound peptides to assess cross-linking efficiencies.

### Data analysis–shortlisting candidate spectra for DSVTRQKEPRAPW bound to ligands of unknown mass

An in-house analysis pipeline was set up to extract candidate spectra potentially originating from an unknown ligand bound to residue K43 in MR1. All files were converted to.mgf using msconvert and further analyzed by R with rawrr package.[49] All MS/MS were selected as candidates or rejected based on the following criteria: (A) Maximum retention difference to the retention time observed for the unmodified DSVTRQ**K**EPRAPW peptide. Within a 2 h gradient, a cut-off of +/− 15 min was used. (B) Presence of DSVTRQ**K**EPRAPW-specific reporter ions: Reporter ions were selected from DSVTRQ**K**EPRAPW backbone fragments that are not ligand bound and therefore identical in all ligand-bound DSVTRQ**K**EPRAPW spectra. Specifically, the ions series y1-y6 (singly and doubly charged) and the ion series b1-b6 (singly and doubly charged) and the 4 precursor ions p++, p+++, p++(-$NH_3$) and p+++(-$NH_3$) were used as reporter ions. Various cut-off values for number of reporter ions required were used throughout the study and are indicated for the particular experiments. (C) Mass range of ligand: Δ-mass values were calculated for all candidate spectra according to the equation [Δ-mass = observed mass – theoretical peptide mass]. This provides information about the attached mass of the ligand, which in turn can be corrected for the mass of the free ligand by increasing the Δ-mass by the mass of one oxygen atom. Cut-off values of Δ-mass >40Da were used to evaluate ligand bound DSVTRQ**K**EPRAPW spectra and cut-off values of 0 > Δ-mass <3.5Da were used to evaluate free DSVTRQ**K**EPRAPW spectra. Spectra fitting these criteria were further shortlisted visually based on the criteria: noise levels, visual quality of the peptide sequence ladder, similarity to spectra observed for free DSVTRQ**K**EPRAPW.

### Mass error estimation for Δ-mass values

When calculating Δ-mass values, we used the measured mass of DSVTRQ**K**EPRAPW crosslinked to the ligand and subtracted the theoretically calculated mass of the unmodified DSVTRQ**K**EPRAPW. This approach led to error propagation effects in the accuracy of determining the mass accuracy of the Δ-mass value. While the mass accuracy of the unknown precursor ion was defined by the instrument specific mass accuracy specifications (e.g., 5ppm for our orbitrap based system), the subsequent subtraction of the theoretically determined mass of the peptide moiety resulted in the absolute amount of the mass error being linked to the unknown Δ-mass values. Therefore, to account for the overall measurement accuracy of the instrument reflected in the modified peptide, varying mass accuracies were considered for the calculated Δ-masses. This effect becomes more prominent for very small Δ-mass and less so for larger modifications, resulting in a dynamic effect of acceptable mass deviation. Acceptable ligand mass error tolerances as a function of ligand size were determined by computing error propagation of various ligand sizes depending on various precursor ion accuracies (5ppm vs. 20ppm, Figure S4A).

### Benchmarking of data analysis pipeline on MM909.24 cells

Intrinsically, our strategy of scouring every MS/MS spectra for the presence of a set of reporter ions and matching a theoretical Δ-mass value was not dependent on an ion originating from modified DSVTRQ**K**EPRAPW. We therefore defined a set of quality

thresholds based on various parameters (such as number of reporter ions, mass deviation, RT deviation) to separate plausible candidates from false positives. Depending on the experiment, we reasoned that we would deal with several main DSVTRQ**K**EPRAPW species: (1) DSVTRQ**K**EPRAPW with no modification, (i.e., originating from unloaded MR1 or MR1 loaded with non-Schiff base bounded ligand), (2) DSVTRQ**K**EPRAPW carrying an Ac-6-FP modification at K43 (pulsed MM909.24 cells), (3) DSVTRQ**K**EPRAPW carrying an unknown modification at K43.

We reasoned that our pipeline should identify DSVTRQ**K**EPRAPW with no modification if Δ-mass value are kept close to zero. In our Ac-6-FP pulsed MM909.24 sample, we first focused our attention on the set of MS/MS spectra reporting a Δ-mass value in the range of $-0.05$ and 4.05 Da as we expected this set to contain MS/MS generated from unmodified DSVTRQ**K**EPRAPW ions (including all ions from the monoisotopic mass up to M+4). Additionally, an arbitrary cut-off of 4 required as the minimal number of reporter ions was used to avoid including random matches. This served as positive control and allowed to determine the number of reporter ions usually being present. 21 MS/MS spectra contained $\geq$4 reporter ions, with high quality spectra predominantly originating from triply charged monoisotopic precursors (Table S3A).

We further isolated the set of MS/MS spectra associated with the Ac-6-FP linkage. Again, we isolated a set of masses covering various isotopes. We calculated the accuracy of the Δ-mass that would have been calculated as an unknown based on the known mass of Ac-6-FP. Assuming an overall instrument specific mass accuracy of 5ppm we deduced a roughly 40-45ppm mass accuracy for the mass range of Ac-6-FP according to the theoretical error propagation calculations described above. Again, monoisotopic precursor ions gave rise to higher quality spectra, with the top hit containing 11 reporter ions and a ligand mass error of < 2ppm (Table S3B). Interestingly, this is lower than we would have assumed from our error propagation assessment, where an overall LC-MS measurement accuracy of 5ppm, would have resulted in a remaining mass accuracy of 40-45ppm for the Ac-6-FP ligand mass. An analogous analysis was performed for the ligand 6-formylpterin (Table S3C).

### Shortlisting candidate compounds for observed Δ-mass values

CEU Mass Mediator was used to transfer Δ-mass values into candidate lists. This step is based on matching the calculated molecular mass of the free ligand candidates to various compound databases (including KEGG, Metlin, HMDB, a.o.). Due to error propagation, compounds were considered as candidates for mass errors of up to 200ppm, however, these candidate lists were subsequently filtered according to the computed error propagation tolerances determined for 5ppm precursor ion tolerance. In practice, this meant using mass tolerance cut-offs ranging from 30 to 60ppm (Tables S2 and S4). Candidates were further shortlisted based on extensive literature research.

### Testing upregulated MR1 surface expression for selected compounds

Ac-6-FP (Santa Cruz Biotechnology), Pyridoxal hydrochloride (Sigma-Aldrich) and Pyrodixal-5'-phosphate (Fluorochem) were reconstituted in DMSO at a concentration of 100 mg/mL and incubated with various target cells at a final concentration of 100 μg/mL in 200 μL of R10 media in a 96U-well plate at 37°C and 5% $CO_2$. After overnight incubation cells were washed, counted and prepared for flow cytometry analysis as indicated above.

### CD69 assays

For CD69 assays, 5 x$10^4$ TPR Jurkat cells and 1 x$10^5$ target cells were plated in 96 U-well plates in 200 μL of R10, then incubated overnight. MR1 ligands; Ac-6-FP, Pyridoxal hydrochloride, Pyrodixal-5'-phosphate (as above) and 5-amino-6-D-ribitylaminouracil (5-A-RU, TargetMol Chemicals Inc. reconstituted to 100 mg/mL in $H_2O$) were added directly to the assays wells to give desired concentrations. CD3/CD28 Dynabeads (2 μL per well) (ThermoFisher Scientific) or 0.02 μg/mL Phorbol 12-Myristate 13-Acetate (PMA) (Promega, Wisconsin, USA) were used as positive controls for TCR-transduced and untransduced Jurkat cells respectively. Cells were stained with viable LIVE/DEAD Fixable Violet Dead Stain Kit (ThermoFisher Scientific), then the following antibodies: CD8 APC Vio770 (to identify the TPR Jurkat cells) (clone BW135/80, Miltenyi Biotec), rCD2 PE (TCR co-marker) (clone OX34, BioLegend), and CD69 APC (clone FN50, BioLegend). Gating for TPR Jurkat cells +/− transduced TCR: (FSC-H versus SSC-H), single cells (FSC-A versus FSC-H), viable cells (SSC-H versus LIVE/DEAD Fixable Violet Dead Stain low/negative), rCD2 versus CD8, then CD69 APC for the mean fluorescence intensity values. Acquisition was performed on a ACEA NovoCyte 3005 with NovoSampler pro (ACEA, Agilent, Santa Clara, CA, USA).

### T107 assay

T cells were rested in R5 (as for R10 with 5% FBS) for 24 h before setting up the assay to help reduce spontaneous activation. T cells (3 x$10^4$) and target (6 x$10^4$) cells were co-incubated for 4–6 h with 30 μM TNF-processing inhibitor (TAPI)-0 (Merck), and antibodies directed against TNF PE Vio770 (clone cA2, Miltenyi Biotec) and CD107a PE (clone H4A3, BD Biosciences). Following co-incubation, cells were washed with PBS then stained with LIVE/DEAD Fixable Violet Dead Stain kit (2 μL per stain of a 1:40 dilution) for 5 min at RT, FcR blocking reagent (Miltenyi Biotec) used according to the manufacturer's instructions (Miltenyi Biotec), and without washing, antibodies against CD3 (clone BW264/56, PerCP conjugated,Miltenyi Biotec), CD8 (clone REA734, APC conjugated, Miltenyi Biotec) and rCD2 (clone OX34, FITC conjugated, BioLegend). Acquisition was performed on a ACEA NovoCyte 3005 with NovoSampler pro (ACEA, Agilent, Santa Clara, CA, USA). Gating was for lymphocytes (FSC-A/H versus SSC-A/H), single cells (FSC-A versus FSC-H), viable $CD3^+$ cells (LIVE/DEAD Fixable Violet Dead Stain low/negative), rCD2+/CD8+, then displayed as TNF versus CD107a.

### Visualization

Visualization was partly done with biorender (https://www.biorender.com/). Spectra and XIC visualization was supported by Skyline.[47]

## QUANTIFICATION AND STATISTICAL ANALYSIS

The results of PEAKS DB searches were filtered using a peptide-level false discovery rate (FDR) of 1% established through parallel decoy database searches. Protein level FDR threshold used to assess MR1 sequence coverage was set to 5%. Reported protein quantifications by PEAKS DB were used for protein abundance plots and assessment of enrichment efficiencies. Skyline was used to visualize XICs of Ac-6-FP bound and unbound peptides and Ac-6-FP. Chromatographic AUC was used for quantification, summing [M], [M+1], and [M+2] ions for peptides and [M], and [M] for Ac-6-FP. Error bars reported denote standard deviation across replicates. Cellular assays were performed in duplicates or triplicates as indicated for each result individually. Bars were used to represent mean with error bars representing standard deviation. For significance testing, we employed one-way Analysis of Variance (ANOVA) using the aov() function from *R* base packages to evaluate differences across groups. Post hoc pairwise comparisons were conducted using Tukey's Honest Significant Difference (HSD) test implemented in TukeyHSD(). Dose response analysis and $EC_{50}$ determination from the flow cytometry data were analyzed using a four-parameter logistic (4PL) model via the drc R package. $EC_{50}$ values and 95% confidence intervals were extracted using ED() function. $R^2$ values were calculated to assess goodness-of-fit.

Cell Reports Methods, Volume *5*

## Supplemental information

# MR1-ligand cross-linking identifies vitamin B6 metabolites as TCR-reactive antigens

**Thierry Schmidlin, Enas Behiry, Hannah Thomas, Garry Dolton, Fabio Marino, Samiul Hasan, Magdalena von Essen, Rose M. Gathungu, Barbara A. Steigenberger, Hayden Selvadurai, Joseph Dukes, Paul E. Brennan, Owen B. Spiller, Jonathan D. Silk, Andrew K. Sewell, and Nicola Ternette**

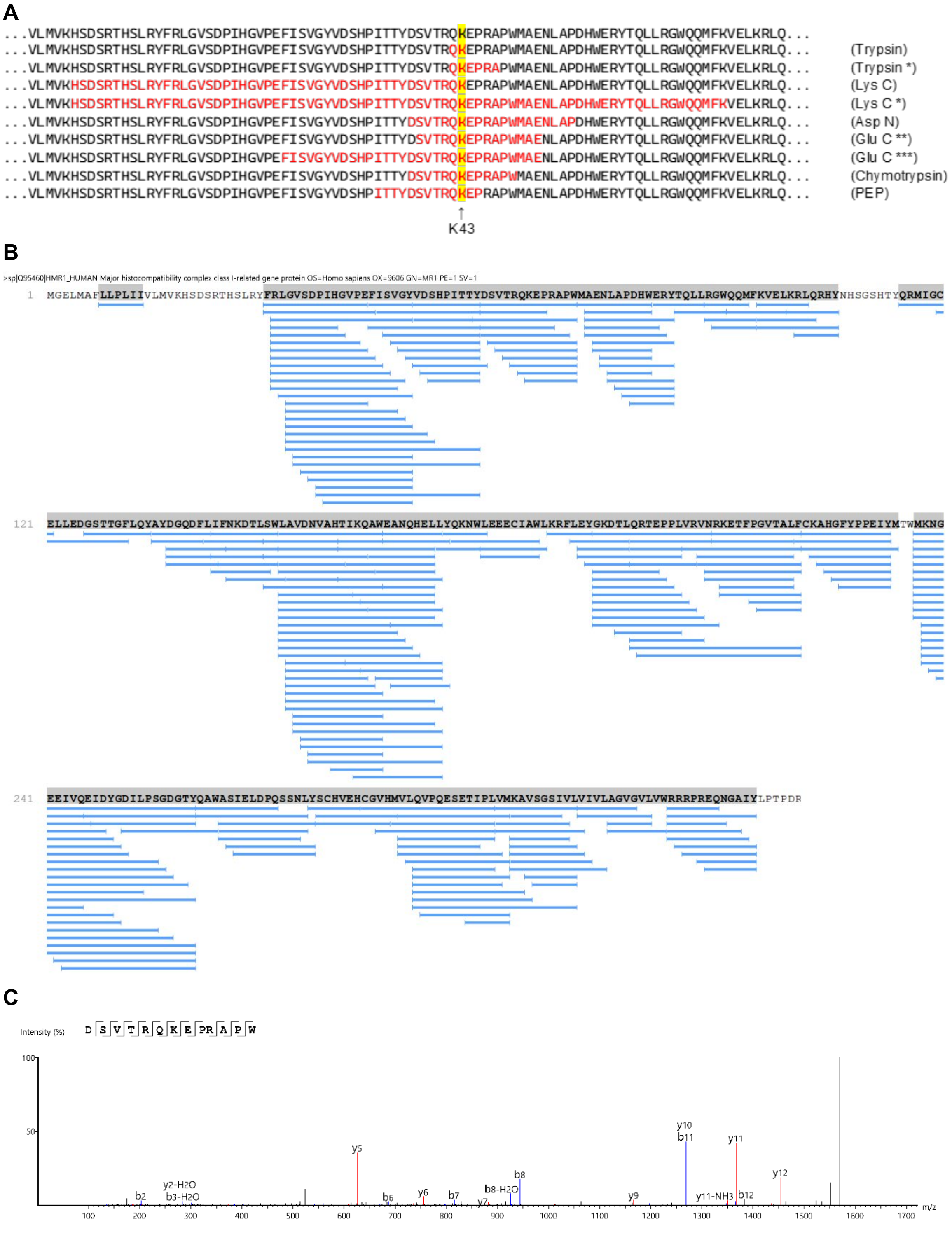

**Figure S1. Chymotrypsin digest of MR1, related to Figure 1. (A)** *In silico* digestion of MR1 around the K43 residue with different enzymes. Lysine 43 (K43) indicated by yellow highlighting. Predicted digestion products with different enzymes (indicated on the right) in red. * denotes assuming missed cleavage at a ligand modified residue. ** denotes digestion performed in phosphate buffer. *** denotes digestion performed in AMBIC. **(B)** Sequence coverage of MR1 in a typical MR1 enrichment experiment digested by chymotrypsin, measured in data-dependent acquisition mode and analyzed by PEAKS DB. Blue bars

represent peptides identified with a <1% FDR cutoff. **(C)** Exemplary PEAKS DB identification as peptide spectra match of DSVTRQ**K**EPRAPW in the same sample. Sequence-specific y-ions are depicted in red and sequence-specific b-ions in blue. Sequence coverage chart above spectrum indicates presence or absence of all sequence-specific y-ions (vertical bar above sequence indicating presence) and b-ions (vertical bar below sequence indicating presence).

A: PEAKS DB identification of unmodified DSVTRQKEPRAPW

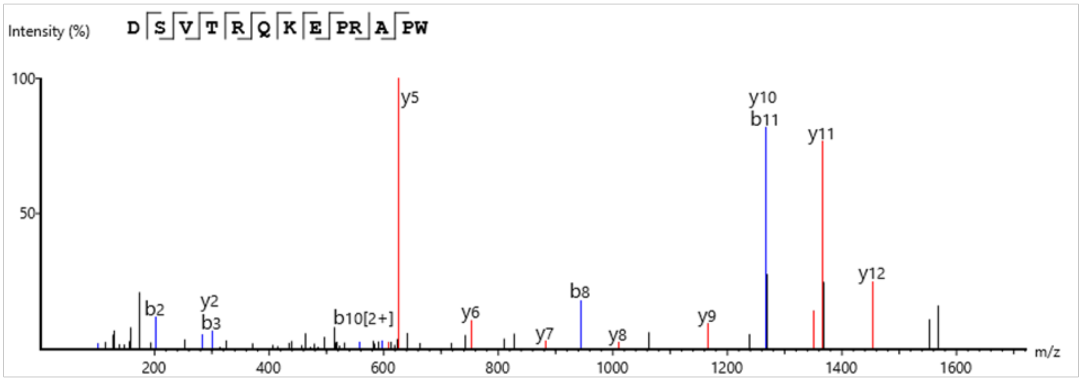


B: PEAKS DB identification of Ac-6-FP linked DSVTRQK(Ac-6-FP)EPRAPW

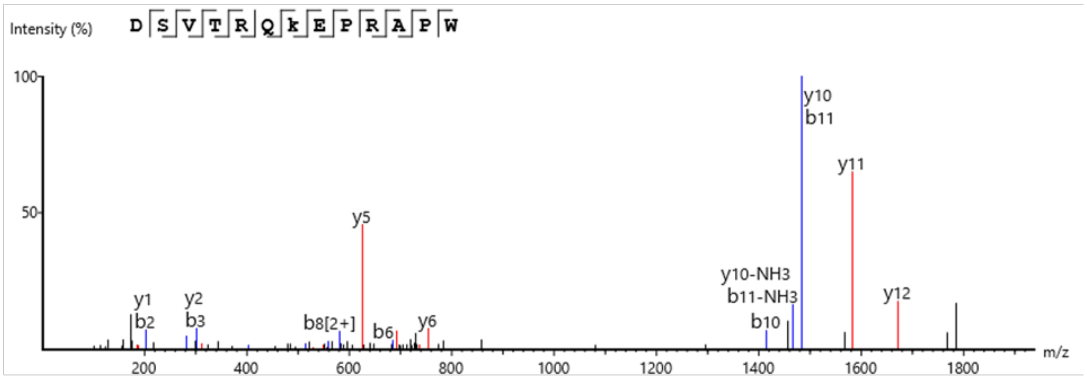


C: Quantitative yield

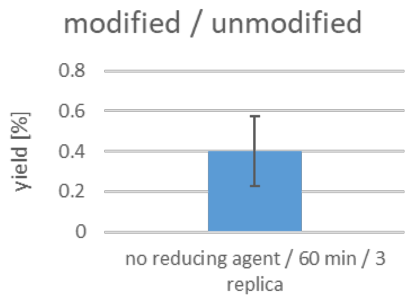


D: Rough yield in the MR1 binding pocket

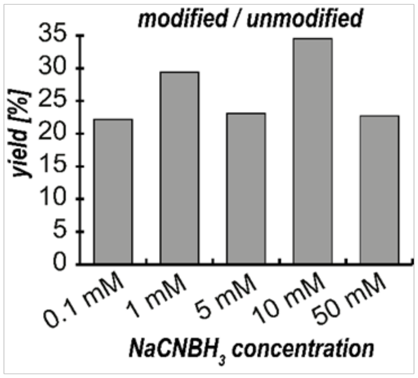


E: PEAKS DB identification of Ac-6-FP linked DSVTRQK(Ac-6-FP)EPRAPW within the MR1 binding pocket

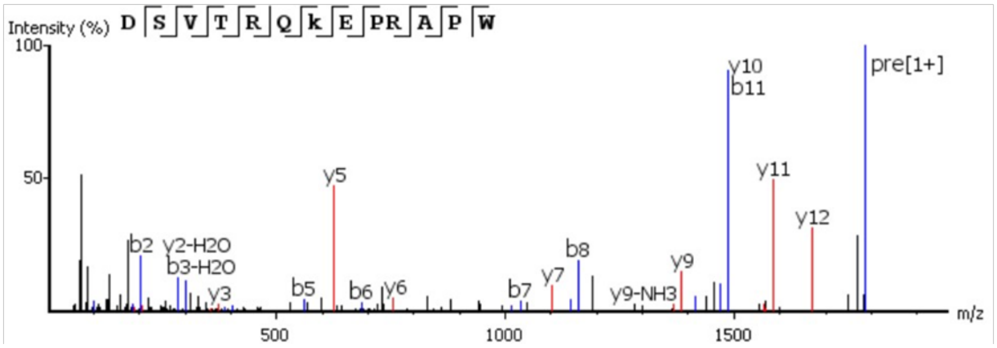

**Figure S2. Reductive Amination of Ac-6-FP with synthetic DSVTRQKEPRAPW peptide in the absence of reducing agent and within the MR1-binding pocket, related to Figure 2. (A-C)** Peptide to Ac-6-FP crosslinking reaction: Results of mixing equimolar amounts of synthetic DSVTRQ**K**EPRAPW and Ac-6-FP incubated at room temperature for 1 h to assess quantitative yield of background reductive amination happening in the absence of $NaCNBH_3$. **(A)** PEAKS DB identification of non-crosslinked DSVTRQ**K**EPRAPW. **(B)** PEAKS DB identification of Ac-6-FP-crosslinked DSVTRQ**K**(Ac-6-FP)EPRAPW (modified residue indicated in lower case). Sequence-specific y-ions are depicted in red and sequence-specific b-ions in blue. Sequence coverage chart above spectrum indicates presence or absence of all sequence-specific y-ions (vertical bar above sequence indicating presence) and b-ions (vertical bar below sequence indicating presence). **(C)** Quantitative yield determined by signal DSVTRQ**K**(Ac-6-FP)EPRAPW / signal DSVTRQ**K**EPRAPW, displayed in percentage. Signal intensity was determined by LC-MS AUC of 3 most intense isotope signals. Error bars depict standard deviation based on 3 replicates. Yields of ~0.4% were substantially lower than yields observed by adding 0.1mM and 1mM $NaCNBH_3$ (related to Figure 2C). **(D-E)** Reductive amination of Ac-6-FP within the MR1-binding pocket with reducing agent: **(D)** Rough quantitative yield of Ac-6-FP cross-linking in the binding groove at five different concentrations of $NaCNBH_3$ performed at $n$=1. The crosslinking product was detected at all tested reducing agent concentrations from 0.1 mM to 24 mM. Estimates for the quantitative yields are in the range of ~20-30% for all conditions. **(E)** Peaks DB identification of Ac-6-FP crosslinked DSVTRQ**K**EPRAPW using *in vitro* refolded MR1/β2M/ligand heterotrimer complex. Sequence-specific y-ions are depicted in red and sequence-specific b-ions in blue. Sequence coverage chart above spectrum indicates presence or absence of all sequence-specific y-ions (vertical bar above sequence indicating presence) and b-ions (vertical bar below sequence indicating presence).

A

MSRSVALAVLALLSLSGLEAIQRTPKIQVYSRHPAENGKSNFLNCYVSGFHPSDIEVDLLKNGERI
EKVEHSDLSFSKDWSFYLLYYTEFTPTEKDEYACRVNHVTLSQPKIVKWDRDMGGGGSGGGGS
GGGGSRTHSLRYFRLGVSDPIHGVPEFISVGYVDSHPITTYDSVTRQKEPRAPWMAENLAPDH
WERYTQLLRGWQQMFKVELKRLQRHYNHSGSHTYQRMIGCELLEDGSTTGFLQYAYDGQDFLI
FNKDTLSWLAVDNVAHTIKQAWEANQHELLYQKNWLEEECIAWLKRFLEYGKDTLQRTEPPLVR
VNRKETFPGVTALFCKAHGFYPPEIYMTWMKNGEEIVQEIDYGDILPSGDGTYQAWASIELDPQS
SNLYSCHVEHCGVHMVLQVPQESETIPLVMKAVSGSIVLVIVLAGVGVLVWRRRPREQNGAIYLP
TPDRWSHPQFEK

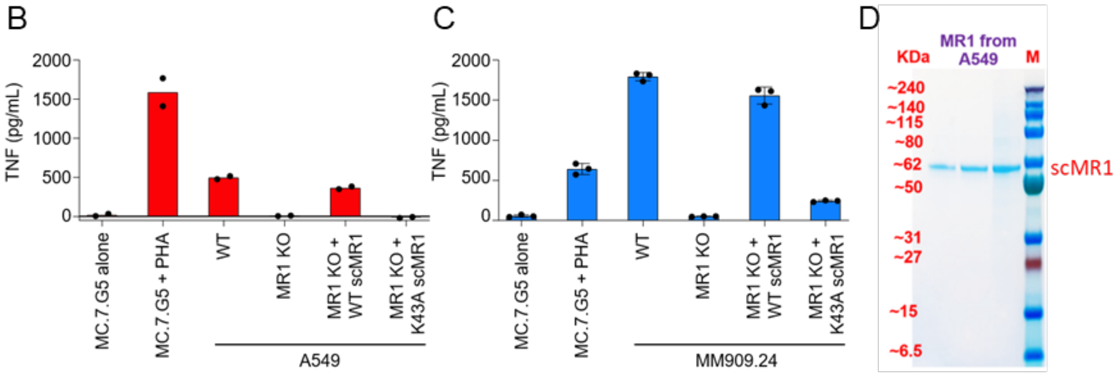


**Figure S3. Design, characteristics and purification of scMR1, related to Figure 3. (A)** To facilitate further enrichment of MR1 we developed a single chain MR1/β2M (scMR1) construct for expression in various cell lines. Our construct was codon-optimized and designed to express the following protein sequence (GGGGS3 linker highlighted in yellow and strep-II-tag in turquoise). **(B-C)** scMR1 presents ligand to the MC.7.G5 cancer-specific MR1-restricted T-cell clone. Overnight activation assay with T-cell clone MC.7.G5 versus various A549 (panel B) and MM909.24 (panel C) cell lines followed by a TNF ELISA, confirming the functionality of the scMR1 constructs. Individual data plots shown for duplicates (panel B) and error bars depicting standard deviation of triplicate conditions (panel C). **(D)** scMR1 can be recovered from A549 cells with high purity. SDS-PAGE showing purified scMR1 from A549 cells transduced with scMR1 as described in the materials and methods. The three samples represent three consecutive FPLC fractions after Strep-Tactin enrichment eluted with 50 mM biotin. M indicates a lane running molecular size marker. MR1 and β2M were also the most abundant proteins purified from scMR1-K43A expressing MM909.24 cells (related to Figure 3D). We concluded that we could recover strep-II-tagged scMR1 proteins from cell extracts with high purity.

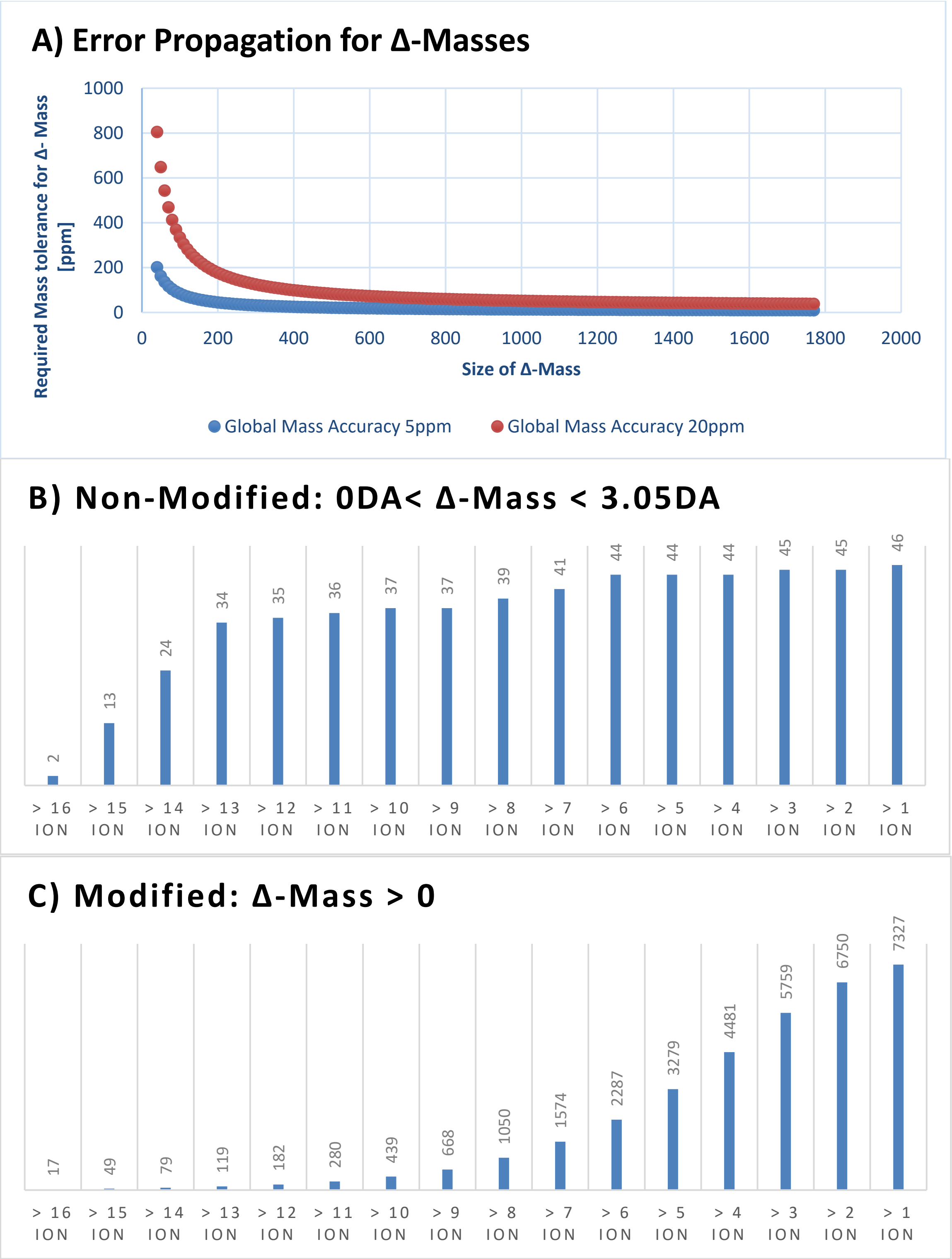


**Figure S4. Mass error propagation effects in respect to ligand size and considerations for reporter ion number cut-off values, related to Figure 4. (A)** Error propagation as a dependency of absolute ligand size: The graph shows the dependency of the accuracy window for Δ-mass values when calculated from the theoretical peptide mass assuming a constant mass accuracy on the precursor ion measurement level. Blue and red dots indicate the error propagation depending the absolute size of the calculated Δ-mass for an

overall mass accuracy of 5ppm and 20ppm respectively. **(B-C)** Assessing reporter ion cut-off values: Cumulative number of MS/MS spectra in four replicates of A549 cells, where a specific number of reporter ions were found. **(B)** Cumulative number of spectra within any of the Δ-mass ranges [0-0.05Da], [0.95-2.05Da] [1.95-2.05Da], [2.95-3.05Da], representing unmodified DSVTRQKEPRAPW and its isotopologues (closed search). We expected this approach to pinpoint all MS/MS spectra of DSVTRQ**K**EPRAPW that were also identified as DSVTRQ**K**EPRAPW in the database search. In this analysis we observed that the number of hits associated with DSVTRQ**K**EPRAPW reached a plateau in the range of 10 -11 reporter ions per spectrum. **(C)** Cumulative number of spectra with a Δ-mass higher than 0Da (open search). Here, the number of hits grew continuously when lowering the required number of reporter ions. The observed discrepancy between open (Δ-mass >3.5 Da) and closed (0 < Δ-mass < 3.5Da) searches was explained by the additional specificity provided by the narrowly defined precursor mass, whereas the open search was prone to be increasingly affected by random matches upon decreasing reporter ion stringency. Hence, the observed plateau could be used to reach an informed choice about reasonable reporter ion number cut-offs which was set to ≥10 ions for subsequent analyses.

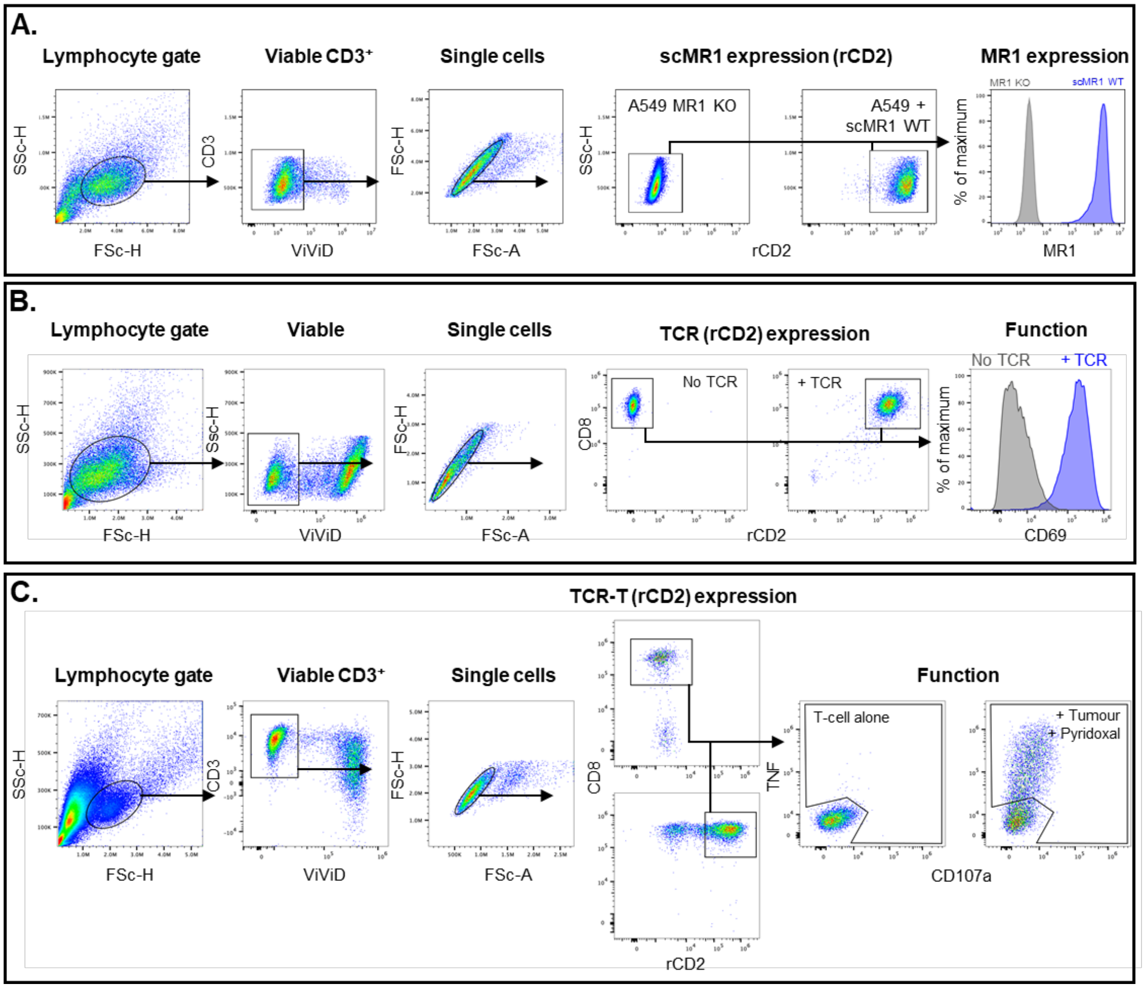


**Figure S5. Flow cytometry gating strategies, related to Figure 5 and Figure 6. (A)** MR1 antibody staining; A549 and A549 MR1 KO cells did not express rCD2 but A549 cells with scMR1 -WT or -K43A were gated on rCD2 as a co-marker, then stained with the MR1 surface antibody. **(B)** CD69 assay; Triple Parameter Reporter (TPR; $CD8^+$) Jurkat cells were gated on $rCD2^+$ for TCR transduced cells then stained with the CD69 surface antibody. **(C)** T107 assay; cells were gated on $rCD2^+/CD8^+$ for A-F7 TCR-T or just $CD8^+$ for the untransduced T cells, then $TNF^+$ versus $CD107a^+$ for reactivity.
